# Focal adhesion kinase-YAP signaling axis drives drug-tolerant persister cells and residual disease in lung cancer

**DOI:** 10.1038/s41467-024-47423-0

**Published:** 2024-05-03

**Authors:** Franziska Haderk, Yu-Ting Chou, Lauren Cech, Celia Fernández-Méndez, Johnny Yu, Victor Olivas, Ismail M. Meraz, Dora Barbosa Rabago, D. Lucas Kerr, Carlos Gomez, David V. Allegakoen, Juan Guan, Khyati N. Shah, Kari A. Herrington, Oghenekevwe M. Gbenedio, Shigeki Nanjo, Mourad Majidi, Whitney Tamaki, Yashar K. Pourmoghadam, Julia K. Rotow, Caroline E. McCoach, Jonathan W. Riess, J. Silvio Gutkind, Tracy T. Tang, Leonard Post, Bo Huang, Pilar Santisteban, Hani Goodarzi, Sourav Bandyopadhyay, Calvin J. Kuo, Jeroen P. Roose, Wei Wu, Collin M. Blakely, Jack A. Roth, Trever G. Bivona

**Affiliations:** 1 Department of Medicine, University of California, San Francisco, San Francisco, CA USA; 2 Helen Diller Family Comprehensive Cancer Center, University of California, San Francisco, San Francisco, CA USA; 3 Department of Cellular and Molecular Pharmacology, University of California, San Francisco, San Francisco, CA USA; 4 Department of Pharmaceutical Chemistry, University of California, San Francisco, San Francisco, CA USA; 5 Instituto de Investigaciones Biomédicas “Alberto Sols”, Consejo Superior de Investigaciones Científícas (CSIC) y Universidad Autónoma de Madrid (UAM), Centro de Investigación Biomédica en Red de Cáncer (CIBERONC), Instituto de Salud Carlos III (ISCIII), Madrid, Spain; 6 Department of Biochemistry & Biophysics, University of California, San Francisco, San Francisco, CA USA; 7 Department of Urology, University of California, San Francisco, San Francisco, CA USA; 8 Department of Thoracic and Cardiovascular Surgery, The University of Texas MD Anderson Cancer Center, Houston, TX USA; 9 Department of Bioengineering and Therapeutic Sciences, University of California, San Francisco, San Francisco, CA USA; 10 Center for Advanced Light Microscopy, University of California, San Francisco, San Francisco, CA USA; 11 Department of Anatomy, University of California, San Francisco, San Francisco, CA USA; 12 Division of Medical Oncology, Cancer Research Institute, Kanazawa University, Kanazawa, Japan; 13 Lowe Center for Thoracic Oncology, Dana-Farber Cancer Institute, Boston, MA USA; 14 University of California Davis Comprehensive Cancer Center, Sacramento, CA USA; 15 Moores Cancer Center, University of California, San Diego, La Jolla, CA USA; 16 Vivace Therapeutics, Inc., 1500 Fashion Island Blvd., Suite 102, San Mateo, CA USA; 17 Chan Zuckerberg Biohub, San Francisco, CA USA; 18 Department of Medicine, Division of Hematology, Stanford University School of Medicine, Stanford, CA USA

**Keywords:** Non-small-cell lung cancer, Cancer therapeutic resistance

## Abstract

Targeted therapy is effective in many tumor types including lung cancer, the leading cause of cancer mortality. Paradigm defining examples are targeted therapies directed against non-small cell lung cancer (NSCLC) subtypes with oncogenic alterations in EGFR, ALK and KRAS. The success of targeted therapy is limited by drug-tolerant persister cells (DTPs) which withstand and adapt to treatment and comprise the residual disease state that is typical during treatment with clinical targeted therapies. Here, we integrate studies in patient-derived and immunocompetent lung cancer models and clinical specimens obtained from patients on targeted therapy to uncover a focal adhesion kinase (FAK)-YAP signaling axis that promotes residual disease during oncogenic EGFR-, ALK-, and KRAS-targeted therapies. FAK-YAP signaling inhibition combined with the primary targeted therapy suppressed residual drug-tolerant cells and enhanced tumor responses. This study unveils a FAK-YAP signaling module that promotes residual disease in lung cancer and mechanism-based therapeutic strategies to improve tumor response.

## Abstract (web summary)

Remaining drug-tolerant persistent (DTP) cancer cells limit the efficacy of targeted therapy in EGFR, ALK and KRAS mutant non-small cell lung cancer (NSCLC). Here, the authors show that focal adhesion kinase (FAK)-YAP signalling supports DTP cells promoting residual disease and targeting this pathway improved tumour response in NSCLC preclinical models.

## Introduction

Lung cancer, of which non-small cell lung cancer (NSCLC) is the most common subtype, is the leading cause of cancer-related mortality worldwide. Comprehensive molecular profiling of NSCLC has defined genetic alterations that drive tumor growth, including somatic mutations in KRAS (32.2 %), EGFR (11.3 %), and NF1 (8.3 %) as well as chromosomal fusion events involving receptor tyrosine kinases (RTKs) such as ALK, ROS1, and NTRK^1^. The development of small molecule targeted agents against these alterations has revolutionized cancer therapy given their improved clinical efficacy and safety profile compared to conventional cytotoxic chemotherapy. Prominent examples of targeted inhibitors used as first-line treatment in NSCLC are Osimertinib and Alectinib for advanced EGFR-mutant or ALK fusion-positive cancers, respectively^2,3^. However, responses to targeted therapies are typically incomplete and residual disease containing slow cycling DTPs remains; tumors ultimately arise with proliferative acquired resistance that drives progression and patients eventually succumb to the disease^4^. Importantly, different molecular programs have been identified in NSCLC patient specimens profiled by single-cell RNA sequencing at residual disease versus at later progression (acquired resistance)^5^. Residual disease cancer cells in NSCLC are characterized by a lineage plasticity switch where adenocarcinoma cells adopt an alveolar cell-like state associated with wound healing and repair, while cancer cells with acquired resistance show an enrichment of invasion- and immune suppression-associated states^5^.

The study of DTPs as a residual disease model has provided important insight into the developmental path of drug resistance^6–9^. DTPs are defined as a small subpopulation of cancer cells that withstand drug treatment by transitioning into a reversible state of no-to-low proliferation and evading drug-induced apoptosis^6–9^. Recent work highlighted the transcriptional co-activator YAP as an important mediator of drug tolerance by limiting pro-apoptotic BMF expression upon targeted treatment in EGFR-mutant NSCLC^10^. YAP and its paralog TAZ are effector molecules operating downstream of the canonical Hippo signaling cascade, which consists of the core MST and LATS kinases that when active inhibit YAP by enforcing its cytoplasmic retention^11^. In addition, YAP activity can also be positively promoted by a complex interplay of other pathways including signaling via SRC family kinases^12^ or Rho GTPases^13^. Upon reduced Hippo signaling or alternative positive signaling input, YAP/TAZ are activated and translocate to cell nuclei where they interact with TEAD transcription factors and regulate gene expression^11^. Previous studies by our groups and other investigators showed that YAP plays important roles in cancer pathogenesis and drug resistance^13–15^. Yet, the complete involvement of YAP in the development of drug tolerance and resistance, the mechanisms by which YAP activation occurs and can restrict therapy response in NSCLC, and the clinical validation of YAP activation in human tumor specimens remain to be fully elucidated. In this study, we elucidate a distinctive mechanism of drug tolerant and residual disease, focusing on the activation of the FAK-YAP/TEAD signaling axis. Additionally, we unveil mechanism-based therapeutic strategies aimed at improving tumor response.

## Results

### DTPs in NSCLC are characterized by reduced proliferation and apoptotic phenotypes

**Fig. 1:**
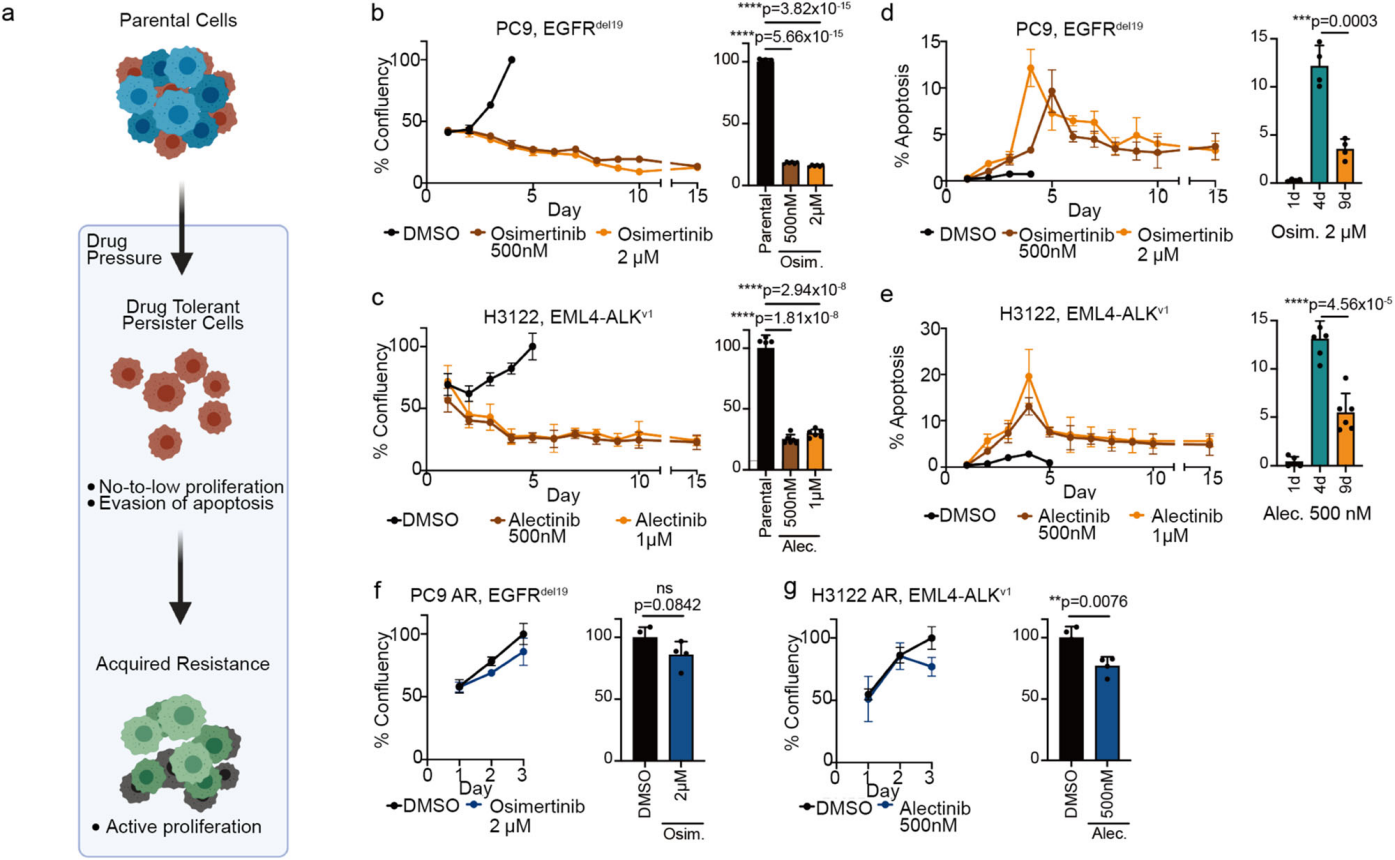
Generation and characterization of drug-tolerant persister cells (DTPs). **a** Schematic highlighting characteristic differences between treatment-sensitive parental cells, drug-tolerant persister cells (DTPs), and acquired resistant cells (AR). A schematic diagram was created with BioRender.com. **b**–**g** High-content microscopy screen monitoring relative cell numbers (**b**, **c**, **f**–**g**) and apoptosis (**d**, **e**) in cells treated with targeted inhibitors. Statistical analysis by *n* = 6 independent experiments, mean ± s.d., two-sided t test. **b**, **c left** Confluency of EGFR-mutant PC9 cells and ALK-fusion positive H3122 cells treated with different doses of osimertinib and alectinib, compared to 0.1 % DMSO control. *n* = 6 independent experiments per data point. **b**, **c**, **right** Comparison of total cell counts in 0.1% DMSO-treated parental cells with the number of DTPs on day 8 was showed in statistical results. Statistical evaluation by two-sided t-test. **d**, **e left** Apoptosis levels in EGFR-mutant PC9 cells and ALK fusion-positive H3122 cells treated with different doses of osimertinib and alectinib, compared to 0.1 % DMSO control. *n* = 6 independent experiments per data point. **d**, **e right** Comparative analysis of apoptosis levels at day 1, day 4 and day 9 of treatment. Statistical evaluation by two-sided t-test. **f**–**g** Confluency of osimertinib-resistant EGFR-mutant PC9-AR cells and alectinib-resistant ALK fusion-positive H3122-AR cells treated with 2μM osimertinib and 500nM alectinib, compared to 0.1 % DMSO control. *n* = 4 independent experiments per data point. The comparison of cell counts in AR cells treated with 0.1% DMSO or the respective targeted inhibitor on day 3 of treatment is shown as a bar graph. Statistical evaluation by two-sided t-test.

We developed several patient-derived preclinical models of residual disease to investigate the underlying mechanisms of drug tolerance. Based on established parameters of drug-tolerant persister cells (DTPs) (Fig. 1a)^6–9^, we evaluated the establishment of a DTP population across cell line models harboring different oncogenic driver mutations (Fig. 1b–e, Supplementary Fig. 1a–g). Cells were treated with their corresponding targeted inhibitor at an 80% inhibitory concentration (IC_80_, Supplementary Fig. 1a–c), according to prior literature on the derivation of DTPs under high-dose drug treatment^6,7,9^. We monitored drug responses across PC9 (EGFR^del19^), H1975 (EGFR^L858R/T790M^), H3122 (EML4-ALK^v1^), H2228 (EML4-ALK^v3a/b^), H358 (KRAS^G12C^), and H1838 (NF1^LOF^) cells. After an initial cytotoxic response (day 1-4) marked by decreased cell culture confluency (Fig. 1b, c, Supplementary Fig. 1d–g) and increased apoptosis (Fig. 1d, e, Supplementary Fig. 1h–j), a subpopulation of cancer cells remained in culture at stable confluency despite continuous drug exposure ( > day 5). These cells represent low-to-non-proliferative and low-apoptotic DTPs, and were detected across all cell line models studied. Notably, DTPs showed a partially reversible phenotype regarding their lack of drug-induced apoptosis, as demonstrated by regained treatment sensitivity after drug washout (Supplementary Fig. 1h–j). Furthermore, suppression of oncogene-mediated signaling was maintained throughout the establishment period of DTPs, while dynamic expression changes and upregulation of known resistance-promoting proteins such as alternative RTKs including FGFR1, ErbB2, and ErbB3 as well as the anti-apoptotic protein Bcl-xL were observed (Supplementary Fig. 2a–c).

Distinct from DTPs, cells exhibiting acquired resistance (AR) following longer-term drug exposure (>6 weeks) were observed to regain their proliferative capacity in the presence of drug (Fig. 1f, g). In addition, significant differences in gene expression were observed by RNA-seq analysis when comparing transcriptional profiles of acutely treated cells (48 hours), DTPs, and cells exhibiting acquired resistance across EGFR-mutant and ALK fusion-positive cancer cell lines under therapy (Supplementary Fig. 2d–f, Supplementary Data 2). This highlights the concept that differential biological events can characterize each distinct treatment phase, and that DTPs show specifically enriched features therein^6,9^.

### Nuclear localization and activity of YAP contributes to drug tolerance in NSCLC

**Fig. 2:**
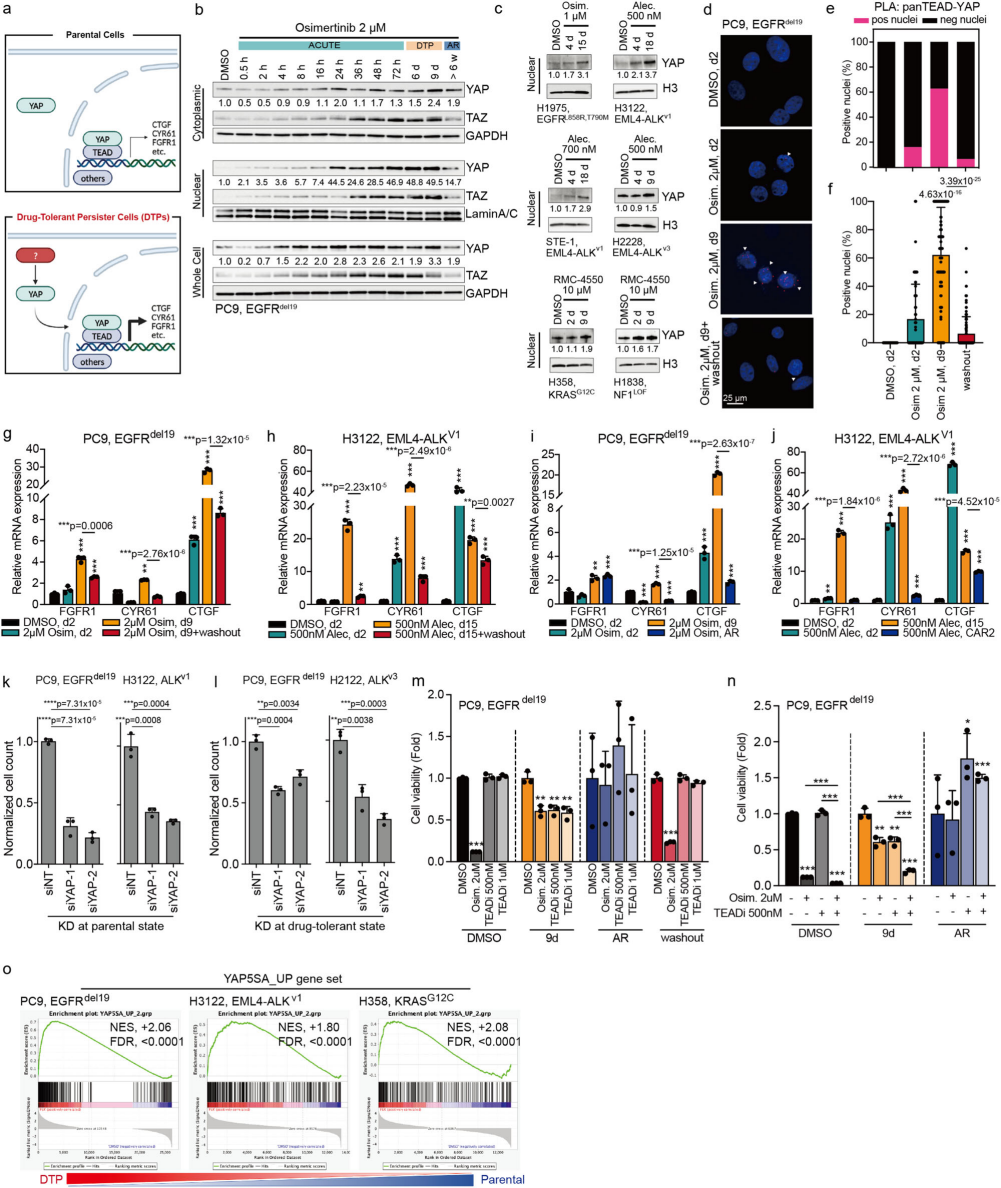
Nuclear localization and function of YAP in DTPs. **a** Schematic representation of YAP nuclear localization and interaction with transcription factors in DTPs. Created with BioRender.com. **b**, **c** YAP levels in nuclear lysates evaluated across PC9 and H1975 cells treated with osimertinib (Osim.), H3122 and H2228 cells treated with alectinib (Alec.), as well as H358 cells and H1838 cells treated with SHP2 inhibitor RMC-4550. Lysates were collected at indicated time points. For PC9 cells, a detailed time course is presented, including corresponding osimertinib-resistant PC9-AR. *n* = 3 independent experiments. **d** PanTEAD-YAP proximity ligation assay (PLA) in PC9 cells treated with osimertinib, analyzed by confocal microscopy. Image is representative of 200 or more cells per condition in total *n* = 4 independent experiments. **e**, **f** Quantification of percentage of cells from (**d**), mean ± s.d., two-sided t test, ***p = 1.08 x 10^-4^ (DMSO vs. Osim.-2d), ***p = 7.32 x 10^-21^ (DMSO vs. Osim.-9d), **p = 0.0023 (DMSO vs. washout), **p = 2.35 x 10^-43^ (Osim.-9d vs. washout). **g**, **h** YAP target genes were significantly induced in PC9 and H3122 DTPs. *n* = 3 independent experiments, mean ± s.d., two-sided t test, (**g**) **p = 0.0028, (**h**) **p = 0.0018, ***p < 0.0001. **i**, **j** YAP target genes were significantly induced in DTP-specific states versus acquired-resistant states in PC9 and H3122 DTPs. *n* = 3 independent experiments, mean ± s.d., two-sided t test, (**i**) **p = 0.0031, (**j**) **p = 0.0002, (**i**, **j**) ***p < 0.0001. **k** Significant decrease in relative DTP cell numbers upon siRNA-mediated YAP knockdown during DTP development in PC9 and H3122 cells. *n* = 3 independent experiments, mean ± s.d., two-sided t test. **l** Significant decrease in relative cell numbers upon siRNA-mediated YAP knockdown in PC9 and H2228 DTPs. *n* = 3 independent experiments, mean ± s.d., two-sided t test. **m** Cell viability of PC9 DTPs was significantly decreased by treatment with TEAD inhibitor VT-104 (TEADi). *n* = 3 independent experiments, mean ± s.d., two-sided t test, **p = 0.0024, **p = 0.0023, **p = 0.0026, ***p < 0.0001. **n** Compared to parental or AR cells, combined treatment with osimertinib and TEADi exerted a significant effect and reduced cell viability, specifically in PC9 DTPs. *n* = 3 independent experiments, mean ± s.d., two-sided t test, **p = 0.0024, **p = 0.0023, *p = 0.0198, ***p < 0.0001. **o** Gene set enrichment analysis (GSEA) for the YAP-5SA_UP gene set (Supplementary Data 3) using RNAseq expression data from untreated parental control compared to PC9 DTPs, H3122 DTPs and H358 DTPs. NES, Nominal Enrichment Score; FDR, False Discovery Rate.

Previous studies conducted by our group and other investigators have suggested a role for YAP in promoting innate and acquired resistance to targeted therapy^16,17^. Recent findings also implicated YAP in drug tolerance and the induction of cancer dormancy in EGFR-mutant NSCLC^10^. These collective findings prompted us to investigate the potential broader role of YAP as a central mediator of drug tolerance and the underlying mechanisms of YAP activation in this context. YAP is a transcriptional co-activator and relocates to the nucleus to modulate gene expression by interacting with specific transcription factors (Fig. 2a)^11^. By performing nuclear-cytoplasmic fractionation assays, we found increased nuclear YAP in DTPs across several cell line models (Fig. 2b, c). As highlighted in PC9 osimertinib DTPs, the upregulation of YAP occurs within the first 24 hours of treatment and nuclear YAP levels peak at the drug-tolerant time point (Fig. 2b). Similarly, a strong increase in nuclear YAP levels was observed in H1975 osimertinib DTPs, H3122, STE-1 and H2228 alectinib DTPs as well as H358 and H1838 RMC-4550 (SHP2 inhibitor) DTPs (Fig. 2c). To independently validate the augmented nuclear YAP present in DTPs, we established isogenic cell lines with an endogenously mNeonGreen-tagged YAP reporter (Supplementary Fig. 3a). Following nine days of osimertinib treatment, a marked enrichment of nuclear YAP was observed (Supplementary Fig. 3a). Given the developmental role of YAP in the control of organ size and its differential regulation by cell density^18,19^, we evaluated nuclear YAP levels in DTPs across different cell confluences (sparse, intermediate, dense) and found increased YAP nuclear localization across all on-treatment conditions (Supplementary Fig. 3b–g). Furthermore, using different matrix stiffness levels to approximate lung solid tumors (25kPa) and non-tumor adjacent tissue (2kPa)^20^, we observed enhanced transcriptional activity of YAP in both matrices following nine days of osimertinib treatment in PC9 cells (Supplementary Fig. 3i). Additionally, cells treated with osimertinib in stiffer conditions exhibited significantly elevated cell survival rates, YAP target gene expression, and nuclear YAP translocation (Supplementary Fig. 3h–j). These results support the idea that YAP translocation and activity occur in response to drug treatment and may be influenced by mechanical forces, consistent with prior literature in other cellular contexts^21,22^.

Canonical YAP signaling involves engagement of TEAD transcription factors^11^. We next expanded our analysis to evaluate the interaction between YAP and TEAD in DTPs. TEAD transcription factors showed a similar nuclear enrichment in PC9 DTPs (Supplementary Fig. 3k). The direct interaction between YAP and TEAD was confirmed by proximity ligation assays (PLA) in PC9, STE-1, and H358 DTPs (Fig. 2d–f, Supplementary Fig. 3l). Similarly, endogenous immunoprecipitation (IP) of YAP validated the YAP-TEAD interaction in PC9 DTPs (Supplementary Fig. 3m). Notably, the nuclear TEAD/YAP interaction was significantly increased by osimertinib treatment and partially reversed upon drug washout in PC9 cells (Fig. 2d–f). To assess the expression of YAP target genes, we conducted qPCR analyzes and observed that the YAP target genes *FGFR1*, *CTGF* and *CYR61* were increased in response to osimertinib and alectinib treatment in PC9 and H3122 cell lines, respectively. Consistent with the PLA results, the qPCR results also showed a significant reduction in YAP transcriptional activity after drug washout (Fig. 2g, h). This reversibility suggests the involvement of a non-genetic mechanism for YAP activation underlying the drug tolerance in these models. To further explore the pivotal role of YAP in the DTP state, we evaluated YAP transcriptional activity in osimertinib-acquired resistance (AR) and acutely treated PC9 cells. Canonical YAP target genes *FGFR1*, *CTGF* and *CYR61* were significantly enriched in the DTP state compared to the acute treatment and AR states (Fig. 2i, j). We next assessed the necessity of YAP activity in DTPs. We observed that genetic silencing of YAP in parental cells suppressed the generation of DTPs (Fig. 2k, Supplementary Table 1) and during the drug-tolerant state reduced the number of DTPs (Fig. 2l, Supplementary Table 1). Moreover, genetic silencing of YAP in PC9 DTPs diminished expression of survival-promoting RTKs (i.e., ErbB2, ErbB3, FGFR1, and FGFR2) as well as the anti-apoptotic protein Bcl-xL (Supplementary Fig. 4a). To further evaluate the involvement of YAP transcriptional activity during the DTP state, we pharmacologically inhibited YAP activity with the established YAP/TEAD inhibitor (TEADi) VT104, one of several analog small molecule inhibitors available with the same mechanism of action against TEAD palmitoylation and consequently YAP/TEAD activity^23^. TEADi-treated cells demonstrated significantly greater responses in the PC9 DTP state than in the acquired resistance and drug washout conditions (Fig. 2m). Additionally, we noticed more pronounced effects when targeted therapy was combined with the TEAD inhibitor in DTPs (Fig. 2n, Supplementary Fig. 4b, Supplementary Table 2). These results underscore a central role of YAP in mediating drug tolerance in targeted therapy treated cells and provide precedence for investigating high-resolution contextual mechanisms of YAP co-activation in future work.

Next, we assessed the sufficiency of YAP in limiting response to initial therapy by expressing the hyperactive form of YAP (YAP-5SA)^24^. This hyperactive form of YAP was sufficient to enhance YAP nuclear localization (Supplementary Fig. 4c) and increase drug tolerance (Supplementary Fig. 4d–k), as well as promote the expression of drug tolerance-relevant YAP targets FGFR2 and Bcl-xL (Supplementary Fig. 4l). Expression of YAP-WT and TEAD transcription factor binding-deficient YAP-S94A showed limited to no changes across different cell line models in terms of treatment response (Supplementary Fig. 4d–l). We next generated a custom gene set of transcripts upregulated in YAP-5SA-expressing PC9 cells (YAP-5SA_UP, Supplementary Data 3) and compared the expression profiles of these genes in parental versus DTPs. There was a significant enrichment of YAP-associated transcripts in DTPs across EGFR-mutant, ALK fusion-positive, and KRAS-mutant cancer cell (Fig. 2o). Moreover, there was a decrease in the YAP-responsive gene expression signature following TEADi treatment in combination with targeted therapy (Supplementary Fig. 4m, Supplementary Data 6). Together, the data suggest that YAP is (hyper-)activated in a conserved manner to promote gene expression changes and cell responses characteristic of drug tolerance in human oncogene-driven lung cancer models.

### Transcriptional adaptation characterizes the emergence of drug tolerance

Non-genetic mechanisms characterizing the development of drug resistance have been highlighted in recent literature^25,26^. To address the involvement of transcriptional adaptation in YAP-mediated drug tolerance, we developed isogenic EGFR-mutant NSCLC cell lines and introduced genetic barcodes of intermediate complexity (totaling 725 barcode groups) to track clonal transition and transcriptional states during the development of the DTP state. We hypothesized that there may be clones that developed after we created monogenic populations due to stochastic events. We utilized group tracing to understand if stochastic processes could create clonal groups that would then impact drug-tolerant phenotypes. To evaluate this concept, we conducted a single-cell RNA sequencing (scRNAseq) trajectory experiment that allowed us to follow cell adaptation under osimertinib treatment (Supplementary Fig. 5a). Early treatment time-points showed signs of apoptosis induction and cell cycle arrest (Supplementary Fig. 5b, c), indicating drug sensitivity. Transcriptional states changed over time, with a bottleneck phase between 8 and 24 h, followed by the emergence of DTPs (Supplementary Fig. 5d). Across the scRNA-seq trajectory in osimertinib-treated cells, there was distinct upregulation of YAP-responsive target genes as defined in the custom signature that we generated using the YAP-5SA hyperactive mutant (Supplementary Fig. 5e). To understand the broader contextual dynamics of YAP activation across transcriptional space, we dissected the YAP gene expression profile into four clusters that were differentially activated across time. Along the trajectory in osimertinib-treated conditions, a unique feature was observed in cluster 3 compared with the DMSO-treated group, as identified through unsupervised hierarchical clustering based on differences in expression patterns at the single-cell level (Supplementary Fig. 5f). This suggests that sub-stratification of these select YAP genes from cluster 3 could serve as important features to consider when understanding responses to targeted inhibitors (Cluster 3, Supplementary Fig. 5e, f, Supplementary Data 4). The latter gene sets showed a correlation in their onset of expression, consistent with prior observations regarding increased YAP nuclear localization within 24 hours after treatment initiation (Fig. 2b) and the defined transcriptional state of developing DTPs (Supplementary Fig. 5d).

By evaluating changes in the composition of genetic barcodes during treatment, no significant enrichment of cell subsets in the trajectory model was identified (Supplementary Fig. 5g). Consistently, comparing genetic shifts in PC9- and H1975-derived isogenic cell lines via whole exome sequencing showed identical density plots of the mutant allele frequency for cells isolated at treatment start (T0) and at drug-tolerance (9 days, Supplementary Fig. 5h, i). These findings align with previous studies, suggesting that the selection of pre-existing subclones is unlikely to be the only basis for the observed phenotype of drug tolerance^27^. Furthermore, bulk RNA sequencing of PC9- and H1975-derived isogenic cell lines validated DTPs-associated phenotypes, such as a reduced cell cycle and an increased YAP-5SA_UP gene signature program (Supplementary Fig. 5j). Thus, the development of drug tolerance and YAP hyperactivation in these models are linked to an adaptive transcriptional plasticity program, which includes distinct features of YAP-responsive gene expression.

### Focal adhesion kinase signaling promotes nuclear localization of YAP during drug tolerance

In canonical Hippo pathway signaling, LATS kinases regulate the subcellular (cytoplasmic versus nuclear) localization of YAP^11^. This regulation is achieved through phosphorylation on specific residues of LATS, including Ser909 and Tyr1079^18,28^. Activated LATS, in turn, phosphorylates YAP on serine residues such as Ser127, leading to sequestration in cytoplasm through binding with 14-3-3 protein^18,28^. Conversely, YAP may undergo nuclear translocation in response to the downregulation of LATS expression or activity through various mechanisms^18,28^. Considering the increased nuclear YAP levels consistently observed across DTPs (Fig. 2b, c), we were prompted to investigate the potential alterations in LATS. We measured the levels of phospho- and total LATS and observed decreased phospho-LATS (Ser909) levels in the PC9 DTPs, while detecting a slight increase in phospho-LATS (Tyr1079) (Supplementary Fig. 6a, b). Intriguingly, despite reduced phospho-LATS (Ser909) levels, there was no loss of phosphorylation of YAP on Ser127 (Supplementary Fig. 6a, b). Notably, the decrease in phospho-LATS (Ser909) levels and the increase in YAP levels were specific to the PC9 DTPs in contrast to parental and acquired resistance conditions (Supplementary Fig. 6b). Moreover, our investigation revealed that LATS knockdown alone was insufficient to promote DTP emergence (Supplementary Fig. 6c) or significant YAP induction in this context (Supplementary Fig. 6d). These findings suggest the possibility that non-canonical regulation of YAP signaling activity may be present in the DTP state.

**Fig. 3:**
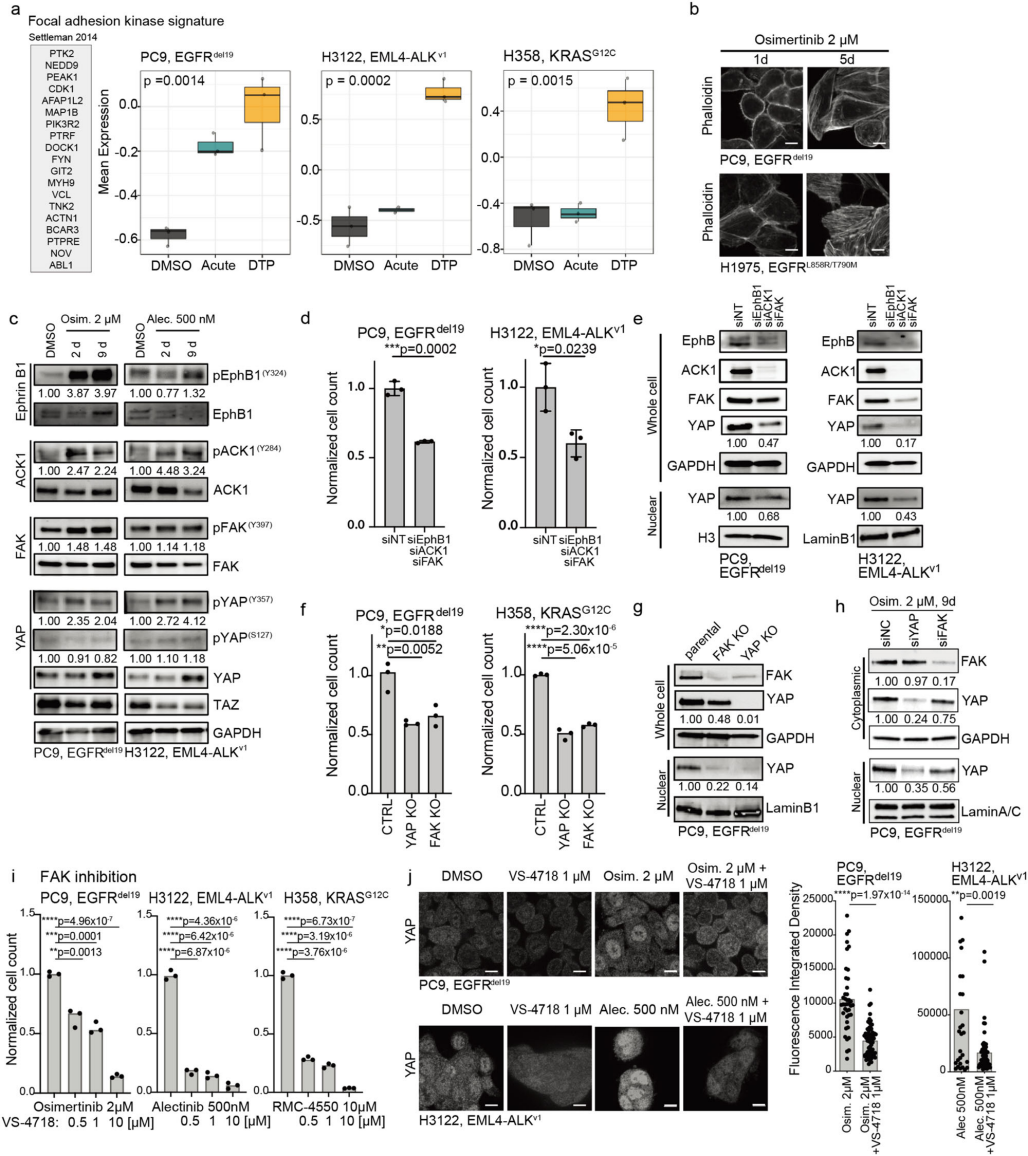
Upstream regulation of YAP by FAK signaling in DTPs. **a** Changes in the FAK expression signature30 in PC9 cells treated with 2 μM osimertinib, H3122 cells treated with 500 nM alectinib, and H358 cells treated with 10 μM RMC-4550. Statistical significance is indicated by two-way ANOVA test, *n* = 3 independent experiments. The box plots display 25th (lower bound), 50th (centre, median), and 75th (upper bound) percentiles, with whiskers (minima (bottom), maxima (top)) extending 1.5 * IQR. **b** Actin cytoskeleton changes upon treatment with 2 μM osimertinib in PC9 and H1975 cells. Image is representative in total *n* = 3 independent experiments, scale bar: 10 μm. **c** Phosphorylation changes of key FAK signaling molecules including EphB1, ACK1, and FAK, as well as for the YAP activating Y357 and inactivating S127 phosphorylation site upon treatment with targeted inhibitors in PC9 cells and H3122 cells. *n* = 3 independent experiments. **d** The relative number of DTPs decreased upon combinatorial knockdown of EphB1, ACK1, and FAK in PC9 DTPs and H3122 DTPs compared to non-target control (siNT). *n* = 3 independent experiments, mean ± s.d., two-sided t test. **e** Decrease in total YAP expression and nuclear localization upon simultaneous knockdown of EphB1, ACK1, and FAK in PC9 DTPs and H3122 DTPs. *n* = 3 independent experiments. **f** The relative number of PC9 osimertinib (2uM) DTPs and H358 RMC-4550 (10uM) DTPs was reduced in cells harboring a CRISPR-mediated FAK or YAP knockout (KO). *n* = 3 independent experiments, mean ± s.d., two sided t test. **g** Changes in YAP expression and nuclear localization upon FAK-KO in parental PC9 cells. *n* = 3 independent experiments. **h** Knockdown of FAK decreased nuclear YAP expression levels in PC9 DTPs. *n* = 3 independent experiments. **i** Normalized DTP numbers upon targeted therapies in combination with FAK inhibitor VS-4718 across PC9 DTPs, H3122 DTPs, and H358 DTPs. *n* = 3 independent experiments, mean ± s.d., two-sided t test. **j** Decrease in YAP nuclear localization in PC9 cells (day 5) and H3122 cells (day 2) upon combined treatment with FAK inhibitor VS-4718 and the backbone targeted treatment; scale bar: 10 μm. Quantification of relative integrated density was performed by automated analysis quantifying the intensity for the protein of interest per nuclei. Image is representative in total n=3 independent experiments, two-sided t-test.

Previous studies identified focal adhesion kinase (FAK) signaling as a central regulator of YAP activity in other cancer cell contexts, offering a non-canonical alternative mechanism for YAP activation and potential clinical intervention using emerging FAK inhibitors^29^. Additionally, the functional relevance of FAK signaling has been underscored in resistance to the first-generation EGFR inhibitor erlotinib in EGFR-mutant NSCLC^30^. Based on these rationales, we hypothesized that FAK could play a role in activating YAP and, consequently, inducing drug tolerance in these oncogene-driven NSCLC systems. We first leveraged an established FAK pathway activation signature^30^ to monitor the progression of drug treatment in EGFR-mutant, ALK fusion-positive, and KRAS-mutant cancer cell lines. A significant increase in FAK signature gene expression during drug treatment was observed, with the highest levels in DTPs (Fig. 3a). FAK gene expression changes correlated with actin remodeling upon drug treatment, resulting in increased actin polymerization and cell elongation (Fig. 3b). These findings are consistent with known roles for FAK in cytoskeletal remodeling^31^. Thus, we evaluated phosphorylation and activation status of FAK, as well as its associated tyrosine kinases within the FAK signature, namely EphB1 and ACK1. It has been previously reported that simultaneous knockdown of all three molecules (likely addressing potential functional redundancies) could induce cell death in EGFR inhibitor-resistant cells^30^. Indeed, an increase in phosphorylation of EphB1, ACK1, and FAK was observed in PC9 and H3122 DTPs (9d, Fig. 3c). FAK has been documented to mediate YAP phosphorylation at Tyr357, leading to an increase in YAP activity^29,32^. Additionally, SRC has been reported as a mediator of direct phosphorylation at specific sites on YAP, including Tyr357, thereby promoting transcriptional activity of YAP^32^. Accordingly, we observed YAP-activating phosphorylation at Tyr357, potentially facilitated through SRC-activating phosphorylation at Tyr416 (Fig. 3c, Supplementary Fig. 6a, b). Moreover, simultaneous knockdown of FAK, ACK1, and EphB1 in DTPs resulted in a significant reduction of cell viability (Fig. 3d, Supplementary Table 4), as well as YAP nuclear localization and overall YAP levels (Fig. 3e). Likewise, CRISPR-mediated FAK knockout (KO) significantly impeded the emergence of DTPs, mirroring the effects observed with direct YAP-KO (Fig. 3f, Supplementary Table 5). Furthermore, FAK-KO PC9 cells showed a significant reduction of YAP nuclear levels (Fig. 3g). Similarly, the siRNA knockdown of FAK in PC9 DTPs caused a reduction in nuclear YAP levels (Fig. 3h). Pharmacological inhibition of FAK signaling by combined treatment with VS-4718, an established potent and selective FAK inhibitor^33^ (Fig. 3i, Supplementary Table 6, Supplementary Fig. 6e, f), or the multikinase inhibitor dasatinib, which targets SRC downstream of FAK^34^ (Supplementary Fig. 6e and g, Supplementary Table 7), in combination with the primary oncoprotein-targeted therapy, lead to a substantial reduction in YAP levels and drug-tolerant cell viability (Supplementary Fig. 6e–g). Notably, we observed limited efficacy of single-agent VS-4718 in parental and AR cells (Supplementary Fig. 6h). We also noted that VS-4718 combined treatment reduced YAP nuclear levels in osimertinib-treated PC9 cells and alectinib-treated H3122 cells (Fig. 3j) and decreased YAP Tyr357 phosphorylation (Supplementary Fig. 6e). Conversely, we further used FAK-KO PC9 cells to overexpress wild-type FAK (WT) and established hyperactive mutants of FAK, including single mutant (Y397D and Y576D) and double mutant (Y397D/Y576D) forms^35^. We uncovered evidence that hyperactive FAK can promote phosphorylation of SRC at Tyr416 and YAP at Tyr357 (Supplementary Fig. 6i). The protein interaction between TEAD and YAP was enhanced by FAK, indicating the active engagement of YAP signaling in the nucleus where YAP/TEAD exert transcriptional effects, although FAK expression did not change overall YAP protein expression levels (Supplementary Fig. 6j–l). The collective findings suggest a critical role for FAK signaling in promoting YAP nuclear localization, potentially with the involvement of SRC, during DTP development in human oncogene-driven NSCLC systems.

### Treatment studies in patient-derived organoid and xenograft models confirm YAP engagement and involvement in residual drug tolerant tumor cells

**Fig. 4:**
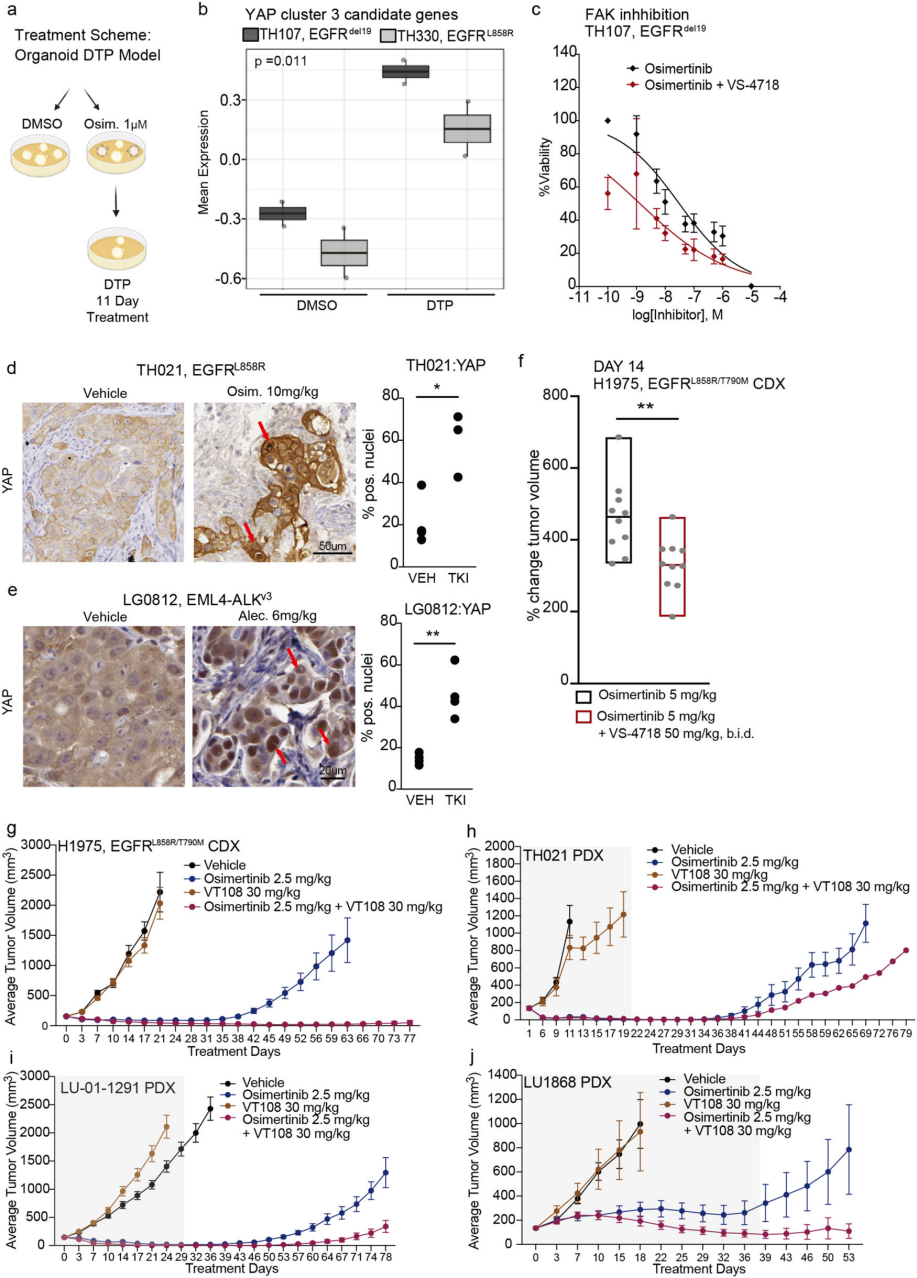
Characterization and therapeutic intervention of residual disease in human NSCLC PDO and xenograft mouse model systems. **a** Schematic presentation for the generation of persister cells in NSCLC patient-derived organoid (PDO) models. Schematic diagram was created with BioRender.com. **b** YAP cluster 3 candidate genes were selected YAP genes based on the time-dependent modulation after 2 uM osimertinib treatment (please see Supplementary Fig. 5e, f). Mean expression of cluster 3 genes in 0.1% DMSO control (DMSO) and drug-tolerant persisters (DTP) across treatment-sensitive EGFR-mutant PDO models, i.e., EGFR^del19^ TH107 and EGFR^L858R^ TH330. Statistical analysis by *n* = 3 independent experiments, mean ± s.d., Statistical significance is indicated by two-way ANOVA test. The box plot displays 25th (lower bound), 50th (centre, median), and 75th (upper bound) percentiles, with whiskers (minima (bottom), maxima (top)) extending 1.5 * IQR. **c** Treatment response to escalating doses of osimertinib upon combinatorial treatment with FAK inhibitor VS-4718 (1 μM) in EGFR^del19^ TH107 DTPs as determined by CellTiter-Glo assay. Statistical analysis by *n* = 3 independent experiments. **d**, **e** Immunohistochemistry staining for YAP in residual tumors cells of EGFR-mutant TH021 and ALK fusion-positive LG0812 PDX models upon treatment with targeted inhibitors (TKI) compared to vehicle control (VEH). TH021 and LG0812 images are representative of total n = 3 and n = 4 independent experiments, respectively. Quantification of nuclear levels (% nuclear) by automated image analysis. Statistical evaluation by two-sided t-test. For TH021: VEH vs TKI, * *p* = 0.0127. For LG0812: VEH vs TKI, ** *p* = 0.0022. Arrows indicate YAP-positive tumor cell nuclei. **f** Relative tumor volume changes in an EGFR-mutant H1975 xenograft (CDX) mouse model across vehicle, 5 mg/kg osimertinib, 50 mg/kg FAK inhibitor VS-4718, and 5 mg/kg osimertinib + 50 mg/kg FAK inhibitor VS-4718 treatment groups. Statistical analysis by *n* = 10 mice. Statistical evaluation by Mann–Whitney U test, **p = ≤0.0021 (two-tailed). The box plot displays the minimum (lower bound), median (centre), and maximum (upper bound). **g**–**j** The combination therapies with 2.5 mg/kg osimertinib and 30 mg/kg TEAD inhibitor VT108 induce more durable response and impair tumor regrowth in (**g**) H1975 CDX model (*n* = 8 mice), (**h**) TH021 PDX model (*n* = 8 mice), (**i**) LU-01-1291 PDX model(*n* = 8 mice) and (**j**) LU1868 PDX model (*n* = 10 mice). For **g** H1975 CDX model, all treatments were continued throughout the entire study. For **h**–**j** PDX models, the gray area indicates the treatment duration, then discontinuation on day 21 for TH021, day 30 for LU-01-1291, and day 38 for LU1868 PDX models.

Patient-derived organoid models can recapitulate determinants of treatment response of patient specimens^36,37^, with recent reports focusing on the development of 3D NSCLC organoid cultures^38,39^. We successfully derived two EGFR-mutant NSCLC organoid cultures from clinical patient specimens and confirmed the presence of the oncogenic EGFR driver mutation (Supplementary Fig. 7a, b). Both EGFR-mutant NSCLC organoid cultures showed sensitivity to treatment with 100 nM osimertinib (Supplementary Fig. 7c). Suppression of EGFR-ERK signaling was verified upon osimertinib treatment (Supplementary Fig. 7d). Thus, these were deemed therapy-responsive and suitable for DTPs generation. We established DTPs derivatives using these organoid cultures (Fig. 4a) and observed an increase in the expression of YAP transcriptional targets in the derived DTPs (Fig. 4b, Supplementary Fig. 7e, Supplementary Data 5). In addition, we demonstrated the sensitivity of the EGFR-mutant NSCLC organoid culture TH107 (EGFR^del19^) to treatment with the FAK inhibitor VS-4718 in combination with osimertinib (Fig. 4c). This extends and corroborates our earlier findings in the cell line-based systems.

We expanded this analysis to encompass in vivo models, utilizing both cell-derived (CDX) and patient-proximate (PDX) xenograft models. This investigation served to further demonstrate that the combination therapies involving FAK or TEAD inhibitors and osimertinib not only induced more profound and long-lasting responses but also impaired tumor re-growth following the discontinuation of drug treatment at the minimal residual disease (MRD) state. To begin, we assessed YAP levels in treatment-sensitive NSCLC PDX samples. We treated EGFR-mutant PDX TH021 (EGFR^L858R^) with 10 mg/kg osimertinib for 7 days and ALK fusion-positive PDX LG0812 (EML4-ALK^v3^) with 6 mg/kg alectinib for 17 days. Both models showed sensitivity to targeted inhibition, resulting in significant regression of tumor volumes under treatment (Supplementary Fig. 8a, b). However, residual tumor lesions remained at the treatment endpoint. Immunohistochemistry (IHC) staining of residual tumor specimens demonstrated increased YAP nuclear levels in residual tumor cells present at the treatment endpoint in both PDX and CDX models (Fig. 4d, e, Supplementary Fig. 8c). Furthermore, RNA sequencing demonstrated a significant induction of YAP-mediated transcriptional changes in inhibitor-treated residual tumors (Supplementary Fig. 8d, e), thus confirming the relevance of our prior in vitro findings and in vivo settings. In addition, combined treatment with VS-4718 alongside the primary oncoprotein-targeted therapy exhibited increased therapeutic efficacy in the EGFR-mutant H1975 (EGFR^L858R/T90M^) xenograft model, without any significant changes in body weight (Fig. 4f, Supplementary Fig. 8f–g).

In addition to inhibiting FAK signaling upstream of YAP, we explored therapeutic strategies to interfere with the YAP/TEAD activation more directly. As previously detailed, canonical YAP-TEAD engagement was notably enriched in DTPs (Fig. 2d–f, Supplementary Fig. 3k–m). TEAD inhibitor treatment in combination with the targeted therapy induced more durable responses at the MRD state in both CDX and PDX models (Fig. 4g–j). There was also a reduction in tumor regrowth after drug withdrawal in the EGFR-mutant PDX model TH021, LU-01-1291, and LU1868 (Fig. 4h–j), with no indication of substantial overall toxicity in preliminary studies (Supplementary Fig. 8h, i). Furthermore, we extended our evaluation of the TEAD inhibitor combined treatment to include multiple resistant CDX and PDX models. Consistent with the results observed in cell line-based studies, we noted limited therapeutic effects in these resistant tumor contexts (Supplementary Fig. 8j–n). In the resistant H1975 xenograft treatment study, the combination therapy with osimertinib and TEAD inhibitor VT108, an *in vivo-*ready chemical analog of VT104 with the same mechanism of action, showed no inhibitory effect on the anti-apoptotic marker *Bcl-XL*, or on the proliferative markers *PCNA* and *KI67* while decreasing YAP/TEAD target gene expression, confirming target inhibition (Supplementary Fig. 8k, Supplementary Table 8). These findings further suggest that the combined treatment was more specifically effective at enhancing response by addressing the YAP-mediated MRD state.

### Humanized murine models confirm YAP-mediated drug tolerance and highlight a role for YAP in modulating treatment-derived changes in the humanized tumor microenvironment

**Fig. 5:**
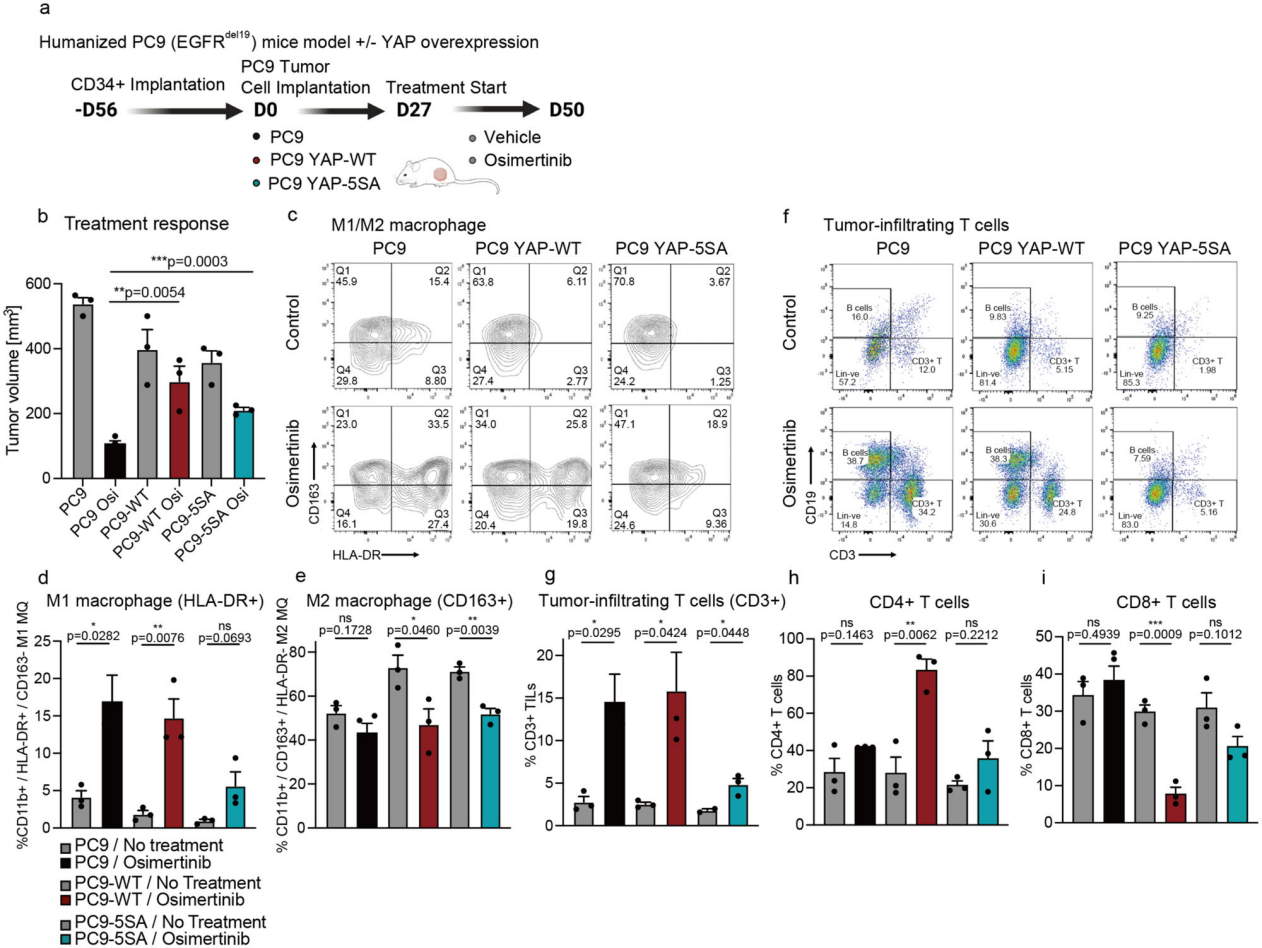
Evaluation of YAP-mediated drug tolerance in immune-competent humanized mice bearing human NSCLC models. **a** Schematic representation of the establishment and treatment study of the humanized EGFR-mutant PC9 xenograft model. A schematic diagram was created with BioRender.com. **b** Changes in tumor volume at treatment endpoint. A Osimertinib [5 mg/kg] treatment study in the humanized PC9 mouse model was conducted comparing parental cells, cells expressing YAP-WT and cells expressing hyperactive YAP-5SA. *n* = 3 mice, mean ± SEM, two-sided t test. **c**–**e** Changes in tumor-infiltrating macrophage populations at treatment endpoint. Macrophage populations are defined as CD11b+ cells, with HLA-DR+ for M1 macrophages and CD163+ for M2 macrophages. *n* = 3 mice, mean ± SEM, two-sided t test. (**c**) The sequential gating strategies are provided in Supplementary Fig. 9d. **f**–**i** Changes in tumor infiltrating T cell populations at treatment endpoint. T-cell populations are defined as CD25+/CD3+ cells, with differentiation of CD4+ and cytotoxic CD8+ T-cells. **f** The sequential gating strategies are provided in Supplementary Fig. 9e. *n* = 3 mice, mean ± SEM, two-sided t test.

Previous studies have highlighted the complex interplay between YAP and the tumor microenvironment (TME), where YAP may both foster tumor cell survival and regulate the immune TME^40^. Thus, we expanded our work to humanized murine models derived from fresh cord blood CD34+ stem cells that show a functional immune cell repertoire in the presence of lung tumor xenograft and PDX models^41^. Implantation of EGFR-mutant PC9 parental cells, YAP-WT, and YAP-5SA overexpressing PC9 cells into humanized mice was performed to establish tumors. We monitored response to osimertinib treatment and found increased drug tolerance was mediated by overexpression of YAP-WT or YAP-5SA (Fig. 5a, b). In addition, an augmentation in nuclear YAP localization was observed in the humanized PC9 mouse model with overexpression of YAP-WT or YAP-5SA (Supplementary Fig. 9a). This confirms previous in vitro results for YAP-mediated effects in the cell line models (Supplementary Fig. 4f–l, Supplementary Table 3) and highlights a similar drug tolerance phenotype in the presence of a functional immune microenvironment in vivo. Of note, important changes in the cellular composition of the tumor microenvironment were observed upon osimertinib treatment. In mice implanted with PC9 parental cells, a treatment-derived increase in tumor-infiltrating myeloid cells and T lymphocytes was observed at treatment endpoint (Fig. 5c–i). Macrophage populations were skewed towards elevated numbers of pro-inflammatory HLA-DR + /CD163- M1 type macrophages upon osimertinib treatment in the PC9 parental tumor cohort (Fig. 5c, d, Supplementary Fig. 9b). By comparison, M1 type macrophages were reduced in vehicle- and osimertinib-treated groups across YAP-WT and YAP-5SA overexpressing cohorts, with the most pronounced phenotype for YAP-5SA overexpressing PC9 cells (Fig. 5c, d, Supplementary Fig. 9b). On the other hand, the abundance of tumor-supportive HLA-DR-/CD163 + M2 type macrophages were increased in untreated groups of YAP overexpressing cohorts compared to PC9 parental tumors (Fig. 5e, Supplementary Fig. 9b). However, osimertinib treatment resulted in a reduction of M2 macrophages and similar abundance across parental and YAP overexpressing groups upon treatment (Fig. 5e, Supplementary Fig. 9b). In addition to changes in the composition of myeloid cell infiltrates, complex alterations in the abundance and phenotype of infiltrating T lymphocytes were observed (Fig. 5f–i). While an increase of infiltrating T lymphocytes was detected upon osimertinib treatment in PC9 parental tumors, lower levels of infiltrating T lymphocytes (YAP-5SA, Fig. 5g) or redistribution of CD4:CD8 T cell ratios in favor of non-cytotoxic CD4 + T cells (YAP-WT, Fig. 5h, i, Supplementary Fig. 9c) were observed upon treatment in YAP overexpressing cohorts. In conclusion, YAP upregulation resulted in a shift towards a more tumor-supportive immune TME, with reduced levels of pro-inflammatory M1 type macrophages and cytotoxic T cells. Thus, modulating YAP/TEAD signaling therapeutically may have dual benefit across the tumor-TME ecosystem in future preclinical studies and potential clinical trials.

### Analysis of NSCLC patient specimens corroborates FAK and YAP activation at residual disease and systematic pharmacological profiling identifies potential therapeutic opportunities

**Fig. 6:**
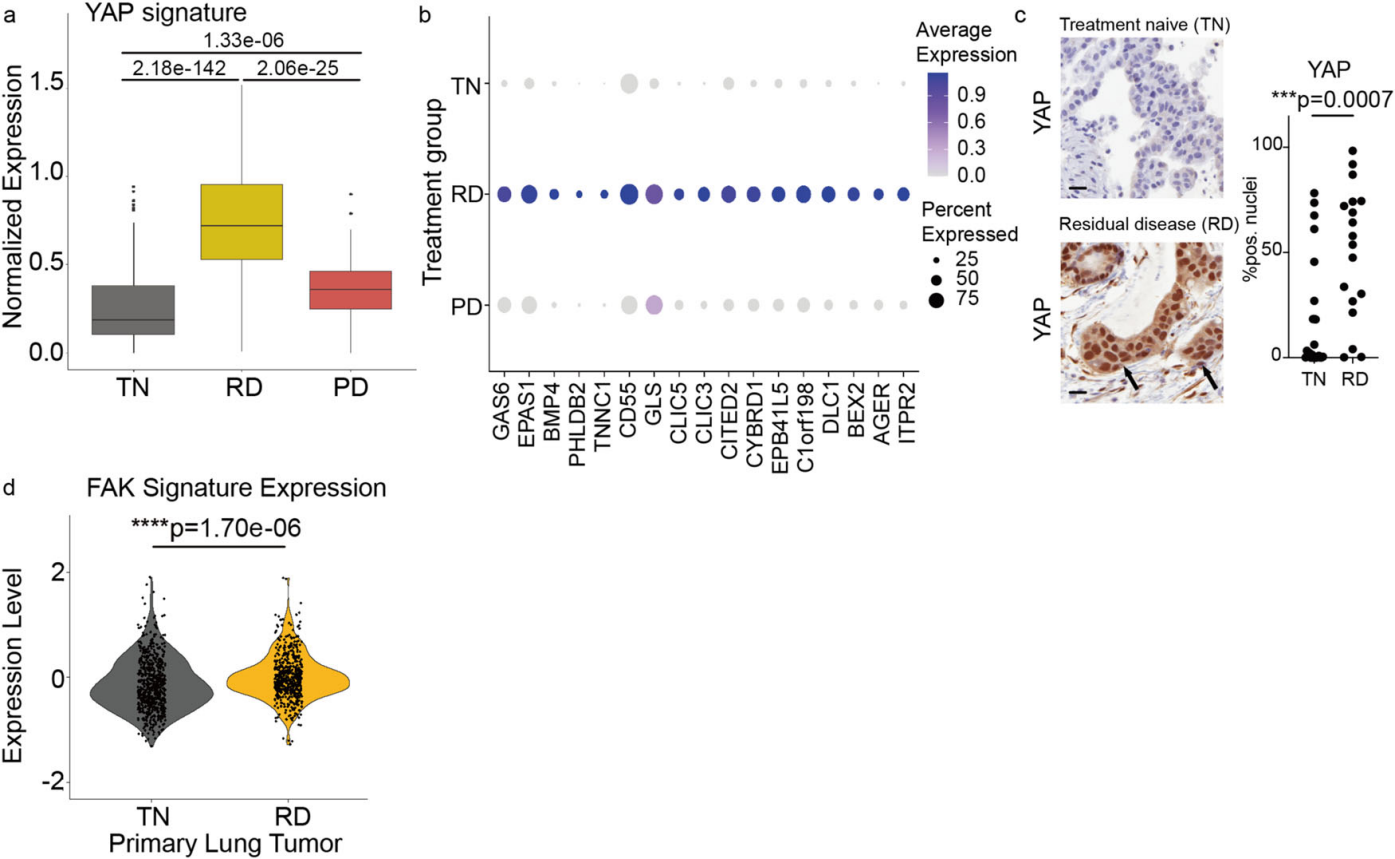
Engagement of FAK and YAP transcriptional programs in patient NSCLC specimens of residual disease. **a** Normalized expression of YAP signature genes across patient specimen classified as TN, RD or progressive disease (PD) using previously published single cell RNA-seq (scRNA-seq) data^1^. The YAP signature determined by genes within the YAP-5SA_UP gene set that are significantly upregulated across PC9, H3122, and H358 DTPs, and were also differentially upregulated in the RD treatment timepoint. The sequencing data were filtered to limit analyzes to malignant lung epithelial cells only (N cells: TN = 621, RD = 484, PD = 138). P-values obtained from two-sided Dunn’s test with Bonferroni adjustment. The box plot displays 25th (lower bound), 50th (centre, median), and 75th (upper bound) percentiles, with whiskers (minima (bottom), maxima (top)) extending 1.5 * IQR. **b** Average expression of individual YAP signature genes highlighted across TN, RD, and PD treatment groups. **c** Immunohistochemistry staining for YAP in patient specimens classified as TKI treatment naïve (TN) or collected at residual disease upon treatment with targeted inhibitors (RD). Quantification of nuclear levels (% nuclear) by automated image analysis. Arrows indicate YAP-positive tumor cell nuclei, scale bar: 20 μm. Statistical analysis by two-sided t-test. *** *p* = 0.0007, *n* = 18 independent experiments. **d** Single-cell RNAseq analysis of clinical samples showed enrichment of the FAK signature in the residual disease state. The significant FAK features include NEDD9, PTPRE, MAP1B, PTRF and NOV. Violin plot data points are single cells’ mean expression of FAK signature. P-values obtained from two-sided Dunn’s test with Bonferroni adjustment.

We next investigated the clinical relevance of our preclinical findings, focusing on FAK-YAP signaling axis in residual disease development in NSCLC. Our previous research highlighted the relevance of scRNAseq profiling in isolating treatment-specific transcriptional programs in NSCLC samples collected from patients at 3 different treatment states: before systemic targeted therapy (TKI naïve, TN), during the residual disease (RD) state, and when tumors had acquired resistance (progressive disease, PD)^5^. We leveraged this scRNA-seq dataset to assess the clinical involvement of YAP transcriptional activity at the RD state. Treatment response states occupy distinct transcriptional space (Supplementary Fig. 10a), with a significant increase in expression of a subset of YAP transcriptional targets in cancer cells present at the RD state compared to TN and PD time-points (Fig. 6a). Similarly, differential transcriptional programs induced upon overexpression of YAP-5SA and in PC9 and H3122 DTPs are more significantly features with the RD state in patients compared to TN and PD time-points, as confirmed by permutation analysis (Supplementary Fig. 10b). In addition, YAP-associated transcriptional targets that are differentially expressed at the RD state in patients (i.e., *GAS6, EPAS1, BMP4, PHLDB2, TNNC1, CD55, GLS, CLIC5, CLIC3, CITED2, CYBRD1, EPB41L5, C1orf198, DLC1, BEX2, AGER* and *ITPR2*) are consistently enriched in the same transcriptional space when considered as a whole gene set (Fig. 6b, Supplementary Fig. 10c). Similarly, IHC confirmed a significant increase of nuclear YAP in tumor specimens at RD state compared to TN samples (Fig. 6c, Supplementary Fig. 10d, Supplementary Data 7). To further evaluate the clinical relevance of the FAK transcriptional signature at the RD state, we analyzed the established FAK signature components (i.e., *NEDD9, PTPRE, MAP1B, PTRF* and *NOV*) in our scRNAseq dataset and confirmed their differential expression at the RD state in the clinical samples (Fig. 6d). These findings mirror our preclinical data and suggest the initial clinical relevance of high FAK-YAP signaling in the RD state in NSCLC patient tumors, an area for future validation in larger clinical cohorts as they become available.

**Fig. 7:**
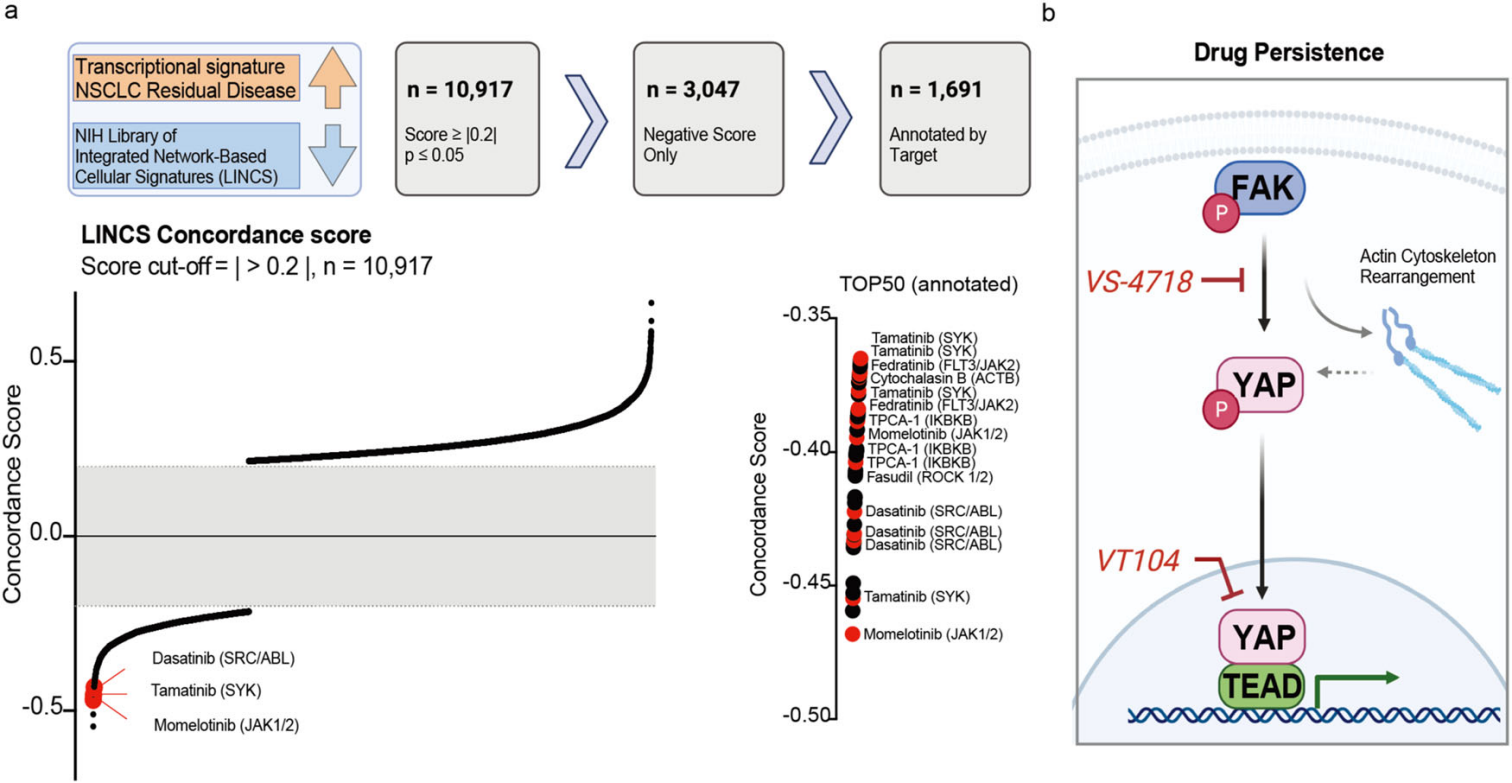
Association of drug-mediated transcriptional changes and expression profiles present in human NSCLCs of residual disease. **a** Concordance score for the correlation of transcriptional scRNAseq profile at the RD timepoint and LINCS L1000 data^44^ collected for drug-mediated expression changes. Schematic of data processing (top) as well as distribution of concordance scores (bottom) are presented. Sigmoidal distribution of concordance scores across drug perturbations showing significant correlation with residual disease-associated expression changes (*n* =10,917). Top 50 negative concordance scores are presented in detail, with inhibitors targeting relevant proteins for focal adhesion / SRC or inflammatory signaling highlighted in red and annotated. A schematic diagram was created with BioRender.com. **b** Pathway schematic for relevant changes in drug-tolerant persister cells, highlighting mechanistic nodes of FAK-YAP-mediated transcriptional adaptation and therapeutic vulnerabilities. A schematic diagram was created with BioRender.com.

Finally, we aimed to generate a systematic framework for identifying pharmacologic agents that could potentially reverse expression signatures identified in RD clinical specimens. This general approach was recently demonstrated in cell line models, with several inhibitors identified that correlated with a reversal of drug-tolerant cell transcriptional changes and were confirmed to show sensitivity upon combinatorial treatment along with the targeted therapy^42^. We used the NIH Library of Integrated Network-Based Cellular Signatures (LINCS)^43^ L1000 transcriptomic platform as a reference dataset for perturbation-induced gene expression changes. This resource includes 71 cell lines and over 20,000 pharmacologic/chemical perturbagens. We identified 10,917 unique drug-cell line combinations with a significant correlation (positive/similar or negative/dissimilar) with clinical NSCLC RD-associated gene expression changes (Fig. 7a, Supplementary Data 8). By selecting for drugs that were associated with opposite expression patterns compared to RD transcriptional profiles (negative correlation, *n* = 3,047) and limiting to pharmacological agents with target information (annotated), we identified over 1,691 drug-cell line combinations that indicate the top candidates for reversal of clinical NSCLC RD-associated expression patterns (Fig. 7a, Supplementary Fig. 11a–c, Supplementary Data 8). Pharmacologic perturbagens that induced gene expression changes that were most significant negatively correlated with the gene expression changes present in RD cancer cells include the JAK1/2 inhibitor momelotinib, SYK inhibitor tamatinib, and SRC inhibitor dasatinib (Fig. 7a, Supplementary Fig. 11b, c). Selective FAK inhibitors within the LINCS L1000 database were limited to PF-562271^44^, which also resulted in a significant negative concordance score (Supplementary Data 8). Similarly, the tankyrase inhibitor XAV-939 showed a negative concordance score and was predicted to reverse NSCLC RD-associated gene expression changes (Supplementary Data 8). This is interesting, as XAV-939 was previously reported to suppress YAP nuclear localization and transcriptional activity^45^ as well as to increase treatment response in EGFR-mutant and ALK fusion-positive upon combination with the primary oncoprotein targeted therapy^5^. Notably, we demonstrated that the FAK inhibitor VS-4718, SRC inhibitor dasatinib, and YAP/TEAD inhibitor treatment showed heightened sensitivity in DTPs in this manuscript (Fig. 3i, Supplementary Fig. 6e–h), providing evidence of the potential general utility of this computational framework for identifying DTP vulnerabilities and strengthening the relevance of FAK-YAP/TEAD signaling and potentially other targetable signaling networks in supporting residual disease.

## Discussion

The preclinical and clinical data we present highlight the relevance of a FAK-YAP signaling axis and for FAK-mediated YAP nuclear localization and activation in contributing to drug-tolerance and residual disease upon treatment with targeted therapy (Fig. 7b). We reveal YAP-driven transcriptional adaptation as a functional mechanism in drug-tolerance, with FAK signaling critically involved in mediating YAP nuclear translocation. Experiments in a humanized mouse model confirmed the role of YAP in reducing treatment sensitivity to targeted therapy and indicated a tumor microenvironment supporting tumor outgrowth. Furthermore, therapeutic vulnerabilities targeting residual cells are identified mechanistically upstream and downstream of YAP. These include inhibiting FAK with VS-4718 and blocking YAP/TEAD with VT104/VT108 (Fig. 7b). Initial clinical relevance of the highlighted mechanisms and YAP activity in RD state were shown by the analysis of unique clinical specimens and a chemical-genetics large-scale dataset analysis to identify drug-based perturbations that highlight potential dependencies that can be exploited to target residual disease, including but not limited to the FAK-YAP/TEAD signaling axis. Importantly, the collective data highlight a role for FAK-YAP/TEAD signaling, more specifically at the residual disease state compared to other treatment contexts, such as acquired resistance, with implications for future clinical trial design and implementation.

Following injury, YAP plays a dynamic role in promoting cell proliferation and regeneration for repair^46,47^. Indeed, studies have shown that YAP is activated in response of injury in normal lung tissue, and we observed a similar injury response signature in clinical samples with DTP cancer cells present at residual disease in our clinical cohort^5^. However, we found that the contribution of YAP to the DTP state is distinct. In DTP cells, YAP protein levels are induced without an increase in cell proliferation. Both in vitro and in vivo models demonstrated increased YAP nuclear localization and transcriptional activity, specifically in the DTP state, but not in the AR state. When we induced cell stress and a slow-cycling state using the CDK inhibitor palbociclib, we observed a no-to-modest increase in YAP protein levels (data not shown), suggesting that not all types of cell stress activate YAP to support drug tolerance. Further understanding when and how YAP functions under conditions of injury, drug tolerance, and drug resistance is important for future studies and for refining treatment strategies.

Clinical approaches targeting the FAK-YAP/TEAD signaling axis are emerging, with recent efforts focused on the development of TEAD inhibitors^10,48^. Inhibition of TEAD S-palmitoylation by small molecules has been reported to impact TEAD function and to block the YAP-TEAD interaction^48^. Optimization of TEAD palmitoylation inhibitors has overcome pharmacological limitations and resulted in in vivo efficacy for VT104 in *NF2*-deficient mesothelioma xenografts^23^. This work provided a rationale for the initiation of the ongoing phase I clinical trial (NCT04665206)^49^, where partial responses in mesothelioma patients have been demonstrated^50^. Similarly, targeting FAK signaling has gained recent momentum, in particular as a combinatorial agent overcoming resistance-associated signaling in cancer therapy^51^. Amongst other contexts, the engagement of FAK signaling has been reported in KRAS-mutant patients treated with the dual RAF/MEK inhibitor VS-6766 in phase I clinical trial (NCT03875820)^51,52^, with subsequent and current clinical studies testing the efficacy of combinatorial treatment with FAK and RAF/MEK inhibitors (NCT04620330, NCT04625270)^53,54^. Our findings provide evidence of FAK-YAP/TEAD signaling engagement in drug-tolerance across EGFR-mutant, ALK fusion-positive, KRAS-mutant, and NF1-mutant NSCLC more broadly and therefore offer rationale for combinatorial treatment approaches testing FAK inhibitors (e.g., VS-4718) and TEAD inhibitors (e.g., VT104/VT108) across several molecularly defined NSCLC subtypes to enhance treatment response to targeted inhibitors against these important oncogenic targets.

FAK inhibitors have shown promising anti-tumor immune effects, and combinatorial treatment of FAK and immune checkpoint inhibition is currently being evaluated in a phase I/IIa clinical study across pancreatic cancer, mesothelioma, and NSCLC (NCT02758587)^51,55^. Our prior work showed significant immune cell alterations at residual disease, characterized by higher T cell infiltrates and the continued presence of pre-dysfunctional cytotoxic CTLA4-positive T cells^5,56^. The FAK-associated tumor-supportive immune microenvironment changes indicate a possible benefit of combinatorial treatment with FAK inhibitors regarding the normalization of an effective immune response at residual disease. Similarly, YAP/TEAD-mediated micro-environmental changes need to be further studied in the respective immune-competent mouse models and could result in additional clinical avenues.

Although YAP can be associated with an epithelial-mesenchymal transition (EMT) and cancer metastasis, we did not find a significant increase of EMT markers in both bulk RNA-seq nor scRNA-seq upon YAP-WT overexpression or expression of hyperactive form of YAP (YAP-5SA). EMT and metastasis may or may not involve YAP engagement in different cancer types and contexts^57^. Moreover, EMT is a complex process that requires intricate signaling interactions and the involvement of various factors. It is likely that YAP activation alone may not be adequate to induce EMT-related genes in this specific context. Further investigation on the systematic regulation of EMT involving YAP and its associated partners is critical for developing treatment strategies aimed at halting tumor progression.

Overall, our findings provide evidence for a distinct mechanism of drug-tolerance and residual disease centered on FAK-YAP/TEAD signaling axis activation that promotes cancer cell survival and potentially tumor immune evasion during targeted therapy. This study highlights the potential for targeting FAK-YAP/TEAD pathway, which hampers the effectiveness of current targeted therapies in oncogene-driven NSCLCs. It also underscores the significance of biological communication between FAK-YAP signaling in cancer cells, and the TME. Our findings have implications for improving treatment regimens to thwart tumor progression by targeting residual disease to enhance clinical responses in oncogene-driven NSCLC patients.

## Methods

### Cell lines and culture reagents

PC9 and derived isogenic cell lines, NCI-H1975 and derived isogenic cell lines, NCI-H3122, NCI-H2228, STE-1, NCI-H358, and NCI-H1838 cells were grown in RPMI medium 1640 supplemented with 10% (v/v %) FBS, 100 IU/mL penicillin, and 100 μg/mL streptomycin. 293 T cells were grown in Dulbecco’s Modified Eagle Medium (DMEM) supplemented with 10% (v/v %) FBS, 100 IU/mL penicillin, and 100 μg/mL streptomycin. All cells were maintained at 37 °C in a humidified atmosphere at 5% CO2.

### Drug-tolerant persister cells (DTPs) generation

For DTPs generation and subsequent analysis by immunoblotting or RNA sequencing, 5 × 10^5^ cells were seeded in 10 cm culture dishes. For DTPs generation and subsequent analysis by confocal microscopy or PLA assay, 5 × 10^4^ cells were seeded in 35 mm glass-bottom dishes (MatTek Corporation). After incubation overnight, cells were treated with an IC_80_ concentration of the respective targeted inhibitor. Treatment was replenished every 3–4 days. DTPs were harvested as indicated and after ≥7 days of drug exposure. For comparison, parental cells were treated for 48 h with 0.1% DMSO (DMSO) or with an IC_80_ concentration of the respective targeted inhibitor (ACUTE). Acquired resistant cell lines were maintained in the drug directly after seeding and treatment was replenished for 48 h with an IC_80_ concentration of the respective targeted inhibitor (AR or RESISTANT).

### Antibodies

For Western blotting, antibodies for phospho-ACK1 (Y284, #3138), Bcl-xL (#2764), phospho-EphB1 (Y324, #3481), EphB1 (#3980), ErbB2 (#4290), ErbB3 (#4754), phospho-FAK (Y397, #8556), FAK (#3285), FGFR1 (#9740), Histone H3 (#9715), Lamin B1 (#12586), phospho-LATS1 (T1069, #8654), LATS1 (#3477), phospho-SRC (Y416, #2101), SRC (#2108), phospho-YAP (S127, #13008), and YAP/TAZ (#8418) were purchased from Cell Signaling Technology. The antibody for phospho-YAP (Y357, #62751) was purchased from Abcam. Antibodies for ACK1 (#sc-28336), FGFR2 (#sc-6930), and GAPDH (#sc-365062) were purchased from Santa Cruz Biotechnology. The antibody for β-actin (#A2228) was purchased from Sigma-Aldrich. Antibodies were diluted according to the manufacturer’s recommendations. For confocal analysis, PLA, and immunoprecipitation, pan-TEAD (#13295) and YAP (#12395) antibodies were purchased from Cell Signaling Technologies and diluted 1:100. For immunohistochemistry, the antibody for YAP (clone H-125, #sc-15407) was purchased from Santa Cruz Biotechnology and diluted 1:150. For immune cell profiling in humanized mice models, fluorochrome-conjugated monoclonal antibodies to the following human antigens were used: CD45-Alexa Fluor 700 (clone 2D1, HI30), CD45-phycoerythrin (PE; clone 2D1, HI30), CD3-PerCp/cy5.5 (clone HIT3a), CD19-PE-cyanine 7 (clone HIB19), CD8-allophycocyanin-cyanine 7 (clone RPA-T8, HIT8a), CD4-Pacific blue (clone OKT4), HLA-DR-PerCp/cy5.5 (clone LN3), CD11b-PE-Cy7 (clone 1CRF-44) (Thermo fisher), CD25-APC (clone CD25-4E3), CD163-APC (clone ebioGH1/61; Thermo fisher). A mouse CD45-FITC (clone 30-F11) antibody was used for gating out murine leukocytes. Most antibodies were purchased from BioLegend, if not otherwise mentioned.

### Pharmacologic agents

Osimertinib (AZD9291), alectinib (CH5424802), ARS-1620, trametinib (GSK1120212), VS-4718 (PND-1186), and dasatinib were purchased from Selleck Chemicals. The TEAD inhibitor VT104 and VT108 were kindly provided by Vivace Therapeutics, Inc. The SHP2 inhibitor RMC-4550 was kindly provided by Revolution Medicines, Inc.

### High-content microscopy screening

Cell lines were seeded in 96-well assay clear-bottom microplates at a density of 2500–5000 cells per well in a total volume of 90 μL per well and incubated at 37 °C, 5% CO2 overnight. Following drug exposure, cell confluency was measured by staining with Hoechst 33342 (Thermo Fisher Scientific) nuclear dye; apoptosis was measured using YO-PRO-1 early apoptosis dye (Thermo Fisher Scientific) and analyzed using a CellInsight High-Content Microscope (Thermo Fisher Scientific) at the indicated time points.

### Apoptosis analysis

Apoptotic cell death was detected by flow cytometry using Annexin V and 7-amino-actinomycin (7-AAD) staining. Cells were harvested and resuspended in Annexin V-binding buffer containing 10% Annexin V-FITC and 10% 7-AAD staining solution (Thermo Fisher Scientific). After an incubation time of 15 min at 4 °C, stained cells were analyzed by flow cytometry.

### Western blot analysis

Whole-cell lysates were prepared by using radio-immunoprecipitation assay buffer (RIPA) [10 mM Tris·HCl (pH 8.0), 1 mM EDTA, 0.1% sodium deoxycholate, 0.1% SDS, 140 mM NaCl] supplemented with protease inhibitor and phosphatase inhibitor (Roche). Nuclear-cytoplasmic extracts were prepared using 0.1% NP-40 in PBS supplemented with protease inhibitor and phosphatase inhibitor (Roche) as previously described^58^. Whole-cell and nuclear lysates were clarified by centrifugation at 17,000 x *g* for 15 minutes at 4 °C. Lysates were quantified using the Pierce BCA Protein Assay Kit (Thermo Fisher Scientific). Equal masses of protein (5−20 ug) were separated by 4–15% of SDS/ PAGE and were transferred onto nitrocellulose membranes (Bio-Rad) for protein blot analysis. After blocking in 5 % milk/ Tris-buffered saline, 0.1% Tween-20 (TBS-T), membranes were incubated with primary antibody overnight at 4 °C, then washed and incubated with secondary antibody for 1 hour at room temperature. Protein bands were visualized using either a fluorescence system (LI-COR) or Amersham ECL chemiluminescent reagent (GE Life Sciences); chemiluminescent signals were visualized with an ImageQuant LAS 4000 instrument (GE Healthcare).

### Generation of endogenously tagged YAP-mNeonGreen2 cell lines

Generation of endogenously tagged mNeonGreen2_1-10/11_ cell lines was performed in EGFR-mutant PC9 cells as described previously^59^ using the sgRNA spacer sequence 5’-AGGCAGAAGCCATGGATCCC-3’. Isogenic cell lines (1-E7 and 2-G10) were generated by single-cell sorting via fluorescence-activated cell sorting (FACS) and outgrowth to stable cell lines. Integration of mNeonGreen2_11_ was confirmed by genomic sequencing and by a reduction in fluorescence upon gene knockdown. Isogenic cell lines showed osimertinib responses equal to parental bulk PC9 cells as evaluated by CellTiter-Glo assay.

### Confocal analysis

Cells were seeded in 35 mm glass-bottom dishes (MatTek Corporation) or µClear 96-well imaging plates (Greiner Bio-One). At the time of harvest, cells were washed carefully and fixed in 4% paraformaldehyde. Cells were then permeabilized in 0.1% Triton-X/PBS and blocked in 5% bovine serum albumin (BSA) / 0.1% Triton-X/PBS. Primary antibody was diluted in 1% BSA / 0.1% Triton-X/PBS and incubated overnight at 4 °C. After washing, secondary antibodies were diluted in 1% BSA / 0.1% Triton-X/PBS and added for 1 hour at room temperature. Where indicated, actin filaments were stained with rhodamine-phalloidin (Thermo Fisher Scientific) as described by the manufacturer. After secondary antibody staining, cells were washed and then stained with DAPI solution (1:1000 in PBS, stock 1 mg/mL, Thermo Fisher Scientific). For endogenously tagged YAP-mNeonGreen2 cells, permeabilization, blocking as well as primary and secondary antibody staining were omitted. Cells were imaged at a Yokogawa CSU22 spinning disk confocal microscope using a Plan Apo VC 60X/ 1.4 Oil objective (Nikon Imaging Center, UCSF). Image analysis was done via Fiji ImageJ software^60^. Quantification of relative integrated density for nuclear levels was performed by automated analysis quantifying the intensity for the protein of interest per nuclei. Source code for quantification is available here: https://github.com/fhaderk/NucYAP.git (10.5281/zenodo.10614956)^61^.

### PLA assay

Cells were seeded in 35 mm glass-bottom dishes (MatTek Corporation). Proximity ligation assays were performed using the Duolink In Situ Red Starter Kit Mouse/Rabbit (Millipore Sigma). In brief, cells were washed carefully and fixed in 4% paraformaldehyde. Cells were then permeabilized in 0.1% Triton-X/PBS before blocking. Primary antibodies were added overnight at 4 °C. PLA probes were added for 1 hour at 37 °C before ligation and amplification. After washing and staining cell nuclei with DAPI solution (1:1000 in PBS, stock 1 mg/mL, Thermo Fisher Scientific), cells were imaged at a Yokogawa CSU22 spinning disk confocal microscope using a Plan Apo VC 60X/ 1.4 Oil objective (Nikon Imaging Center, UCSF). Image analysis was done via Fiji ImageJ software^60^ and PLA signals per nuclei were counted.

### Endogenous immunoprecipitation

Primary antibodies were coupled to Dynabeads Protein G beads at a 1:5 ratio (antibody:beads, v/v) by constant rotation for 6 hours at 4 °C. Nuclear fractions of DTPs were prepared using the NE-PER Nuclear and Cytoplasmic Extraction Reagents (Thermo Fisher Scientific) and kept on ice. Lysates were quantified using the Pierce BCA Protein Assay Kit (Thermo Fisher Scientific). Equal masses of proteins (300 µg) were added to antibody-coated beads and incubated by constant rotation overnight at 4 °C. Bead-coupled samples were washed and resuspended in 4x Laemmli buffer [277.8 mM Tris-HCl, pH 6.8, 44.4% (v/v) glycerol, 4.4% LDS, 0.02% bromophenol blue, supplemented with 10% (v/v) 2-Mercaptoethanol]. Beads were collected and samples were analyzed by Western blot analysis as outlined above.

### Knock-down and CRISPR knock-out experiments

CRISPR-mediated YAP and FAK knock-out cells were engineered by Synthego (Synthego Corporation, Redwood City, USA). Transient YAP silencing was achieved by knock-down using individual Dharmacon ON-TARGETplus YAP1 siRNAs (siYAP#1: J-012200-08, siYAP#2: J-012200-07) compared to non-target control (D-001810-02). Knock-down of focal adhesion kinase signature genes *EphB1* (siEPHB1: L-003121-00), *FAK* (siPTK2B: L-003165-00), and *ACK1* (siTNK2: L-003102-01) as well as of *LATS1* (L-004632-00) and *LATS2* (L-003865-00) was induced using SMARTpool Dharmacon ON-TARGETplus siRNAs compared to non-target control (D-001810-10). Target cells were transiently transfected using Lipofectamine RNAiMAX Transfection Reagent (Thermo Fisher Scientific). SiRNA-mediated knock-down was initiated either at the beginning of treatment with the targeted inhibitor (knock-down during DTPs generation) or when DTPs were established after 7 days of treatment (knock-down at DTPs state). In both cases, knock-downs were repeated every three days and a consecutive number of three knock-downs was performed before harvest. Knock-out and knock-down of the protein of interest were verified by Western blot analysis.

### YAP-5SA overexpression

Full-length YAP was amplified by PCR using forward primer 5’-TTTGACCTCCATAGAAGATTCTAGATGGAACAAAAACTCATCTC-3’ and reverse primer 5’-AGCGATCGCAGATCCTTCGCGGCCGCTATAACCATGTAAGAAAGCTTTC-3’ from pQCXIH expression constructs encoding myc-tagged YAP-WT (Addgene #33091), YAP-5SA (Addgene #33093), and YAP-S94A (Addgene #33094), respectively. After XbaI/NotI digestion, PCR products were cloned into the lentiviral pCDH-puro plasmid backbone and correct insertion was verified by Sanger sequencing. For lentivirus production, 293 T cells were co-transfected with pCDH-YAP expression plasmids and lentiviral packaging plasmids pCMV-dR8.91 and pMD2.G using the TransIT-LT1 Transfection Reagent (Mirus Bio). Viral supernatant was harvested 72 h after transfection. Target cells were infected and selected with 1 µg/mL puromycin. YAP overexpression and YAP nuclear localization upon treatment with 0.1% DMSO or 2 µM osimertinib in stable transduced PC9 cells was monitored by Western blot analysis and confocal microscopy.

### Cell viability and DTPs quantifications

Cell survival and DTPs numbers upon genetic or pharmacologic perturbations were evaluated by counting cells using a Vi-CELL XR Cell Viability Analyzer (Beckman Coulter, Inc.). Raw counts for counting cells using a Vi-CELL XR Cell Viability Analyzer are presented in Supplementary Tables 1–7. Response to escalating drug doses in stable transduced PC9 and H358 cells was analyzed by CellTiter-Glo assay (Promega). For the latter, cells (5 × 10^3^/well) were seeded in clear-bottom 96-well plates. After overnight incubation, cells were treated with escalating drug concentrations and harvested at day 5 post drug treatment.

### RNA sequencing and gene set enrichment analysis

RNA was extracted from snap-frozen tissue or cell pellets. For tissue samples, tissue was minced using a liquid nitrogen-cooled mortar and pestle before RNA extraction. RNA isolation was performed using the RNeasy Mini kit (Qiagen) including an on-column DNase I digestion. RNA quality was assessed by automated electrophoresis using the RNA 6000 Pico Kit and an Agilent 2100 BioAnalyzer (Agilent Technologies, Inc.). RNA was quantified using the Qubit RNA HS Assay Kit and a Qubit 2.0 fluorometer (Thermo Fisher Scientific). Library preparation and paired-end 150 bp (PE150, Illumina) RNA sequencing was performed by Novogene (Novogene Corporation, Sacramento, USA). RNA-Seq reads were mapped to the hg19 reference genome using STAR (Spliced Transcripts Align to a Reference, v2.4.2a). The expression level of transcript per million (TPM) reads were quantified using RNA-Seq by Expectation-Maximization algorithm (RSEM v1.2.29). The quantified gene expressions of 26,334 transcripts (including coding genes and non-coding genes) were processed in R studio. Differentially expressed genes between tumor and normal samples were identified using the EdgeR algorithm. Gene set enrichment analysis was done using GSEA 4.0.1 software^62,63^.

### Single-cell derived clone generation

Single-cell clones were from PC9 cells. These clones were cultured for 60 days to generate sufficient material for experimentation, resulting in approximately 45 cell doublings. The cells were transduced with a lentiviral barcoding plasmid called pBA571. This plasmid contains an 18-base pair static barcode downstream of BFP. The total potential barcode combinations were vast, approximately 6.8e10, and we used large-scale bacterial propagation techniques to maintain barcode diversity. After viral particle production and titration, we transduced the PC9 parental populations at a low MOI (~0.1) in T175 flasks, totaling around 15 million cells at the time of transduction. Subsequently, we sorted approximately 1.5 million cells three days after transduction, utilizing BFP as a selection marker. These sorted cells were then serially plated in individual wells of a 6-well plate, with cell numbers ranging from 100 to 5000. The founder populations were allowed to expand for 30 days, after which banks were prepared, and genomic DNA (gDNA) was collected to empirically determine the number of static barcodes present. We employed a two-step custom amplicon preparation method for barcode enrichment from the gDNA, utilizing specific primers for round one amplification:

prmJY18145: ACACTCTTTCCCTACACGACGCTCTTCCGATCTGCACAGTCGAGGCTGAT

prmJY18146: GTGACTGGAGTTCAGACGTGTGCTCTTCCGATCTCCTAGCAAACTGGGGCACAAGC

For the second round of amplification, standard i7/i5 indexing primers for Illumina instruments were used. We selected clones with an initial seeding density of 1000 uniquely barcoded cells in the founder population. To determine the true whitelist number of unique barcode clones within the population, we applied an empirical False Discovery Rate (FDR) cutoff method. Barcodes with the highest read counts were prioritized, and additional barcodes were incrementally included in the low-depth sequencing range. At each grouping of barcodes, we calculated the Hamming distance between them. FDR was estimated by considering barcodes within a Hamming distance of one as false positives, and those beyond a Hamming distance of one as true positives. With an FDR threshold of 0.05, we determined that there were 560 barcodes in the initial founder population of 1000 seeded cells. The cells were expanded for an initial 30 days, totaling 60 days of growth. An estimated ~45 cell doublings occurred during this period. The cells were continuously passaged to avoid bottlenecks. From the single-cell clonal stage to the point of single-cell group tracing experimentation, approximately 150 days elapsed. This timeframe included 60 days for creating the initial single-cell clone, 30 days for the lentiviral transduction workflow, and 60 days for the group tracing bottleneck and expansion of the labeled founder populations. In total, an estimated ~112 doublings occurred. This allowed for a substantial search space ( ~ 2.6e + 33 possible daughter cells) and provided a reasonable model system for studying stochastically determined cell group fates.

### scRNA sequencing trajectory

A BFP-tagged barcode library (Addgene #85968) was delivered via lentiviral infection into isogenic EGFR-mutant PC9-C2 and H1975-B10 cells. Cells were sorted and serially titrated to allow for ~1000 unique barcode groups. After expansion, cells were subjected to 0.1% DMSO or 2 µM osimertinib treatment and frozen down at the indicated timepoints. Cells were thawed, hashed with TotalSeq A anti-human hashtag antibodies (BioLegend), and pooled for single-cell RNA sequencing on the 10X chromium v3 platform (10x Genomics). Cell hash libraries were prepared as specified by BioLegend. Custom barcode amplification was performed by two rounds of PCR. Libraries were sequenced on the NovaSeq Illumina platform (Center for Advanced Technology, UCSF). After NGS sequencing, cells were called with 10X Cell Ranger pipeline and cell hashes were called using the scEasyMode package in Python. In addition, bulk genomic barcodes were prepared from the same time points used for single-cell RNA sequencing using the Quick Extract gDNA extraction protocol (Lucigen Corporation) and custom barcode amplification primers for NGS library preparation. A custom script for calling genomic barcodes mapping between single-cell genomic barcodes and bulk genomic barcodes collected from the same samples was used to assess population frequency and map onto single-cell transcriptomes. The diversity index was calculated as 1 - Sum_i (pi^2), where pi is the relative abundance of lineage i. The diversity index is at its maximum when all barcode groups are equally abundant and decreases if some barcode groups are enriched and others depleted. The index was scaled by the max possible index given the number of barcode groups which is max (Lineage diversity index) = 1 - n[(1/n)^2] = 1 − 1/n; n: number of barcode groups. Source code for scRNA sequencing and genetic diversity assessment is available here: https://github.com/BivonaUCSF/YAP.git (10.5281/zenodo.10632418)^64^.

### Whole exome sequencing

DNA was extracted from snap-frozen cell pellets using the DNeasy Blood & Tissue kit (Qiagen). DNA quality was assessed by automated electrophoresis using the High Sensitivity DNA Kit and an Agilent 2100 BioAnalyzer (Agilent Technologies, Inc.). DNA was quantified using the Qubit dsDNA HS Assay kit and a Qubit 2.0 fluorometer (Thermo Fisher Scientific). Library preparation and paired-end 150 bp (PE150, Illumina) DNA sequencing were performed by Novogene (Novogene Corporation, Sacramento, USA). Pair-end fastq files were mapped to the hg19 genome and mutation calling using the SeqMule pipeline^65^. The VCF files were annotated using ANNOVAR software at a high-performance computing cluster (UCSF Helen Diller Comprehensive Cancer Center). Further analysis of annotated variants was conducted under the RStudio/R environment.

### De-identified patient tumor samples and use of de-identified human tissue

All patients gave informed consent for collection of clinical correlates, tissue collection, and research testing under Institutional Review Board (IRB)-approved protocols (CC13-6512 and CC17-658, NCT03433469) in a de-identified manner. Patient demographics are listed in Supplementary Data 1. Patient studies were conducted according to the Declaration of Helsinki, the Belmont Report, and the U.S. Common Rule.

### NSCLC organoid cultures

Organoid cultures from NSCLC specimens were established as previously described^66,67^. The patients, whose samples were used to generate the organoids, provided informed consent for collection of clinical correlates, tissue collection, research testing under Institutional Review Board (IRB)-approved protocols (CC13-6512 and CC17-658, NCT03433469) in a de-identified manner. Organoid cultures were embedded in Reduced Growth Factor Basement Membrane Extract, Type 2 (BME2) matrix (Thermo Fisher Scientific) and maintained in Dulbecco’s Modified Eagle’s Medium/Ham’s nutrient mixture F12 (DMEM/F-12) GlutaMAX supplement, supplemented with 100 U/mL penicillin/streptomycin, 10 mM HEPES, 25 nM hRspondin, 1x B27, 5 mM Nicotinamide, 1.25 mM N-Acetylcysteine, 500 nM A-8301, 500 nM SB202190, 50 µg/mL Primocin, 100 ng/mL hNoggin, 100 ng/mL hFGF-10, and 25 ng/mL hFGF-7^67,68^. Mutational profiling of organoid cultures was performed by whole-exome sequencing. Signaling alterations upon osimertinib treatment in EGFR-mutant organoids were evaluated by treating single suspensions for 2 hours and subsequent Western blot analysis. Drug sensitivity was analyzed by 3D CellTiter-Glo assay (Promega). In brief, single cells suspensions were prepared by TrypLE digestions, and cells (7.5 × 10^3^/ well) were seeded in BME2 on clear-bottom 96-well plates (Corning). After seven days in culture, newly formed organoids were treated with indicated drug concentrations in reduced growth factor media. Five days after treatment initiation, the viability of cells was assessed. DTPs generation of organoids was performed as outlined, seeding NSCLC organoid cells (1.8 × 10^5^/ well) embedded in BME2 in a 6-well plate format. After three days in culture, organoids were treated with 0.1% DMSO or 1 µM osimertinib. DMSO-treated control cells were harvested three days after treatment. For DTPs, the drug was replenished every 3 days, and cells were harvested post 11 days on treatment. The engagement of YAP signature genes was evaluated by RNA sequencing and gene set enrichment analysis.

### Subcutaneous xenograft and PDX experiments

All animal experiments were conducted under UCSF IACUC-approved animal protocol no. AN187306-01B or according to the guidelines approved by the IACUC of WuXi AppTec or Crown Bioscience, Inc., following the guidance of the Association for Assessment and Accreditation of Laboratory Animal Care (AAALAC). H1975 tumor xenografts were established by injection of one million cells in a 50/50 suspension of matrigel/PBS into 6- to 8-wk-old female SCID mice for investigating the FAK inhibitor VS-4718, or female BALB/c nude mice for studying the TEAD inhibitor VT108. In the case of FAK inhibitor VS-4718-treated models, once the tumors reached an average size of ~200 mm3, mice were randomly assigned to receive treatment with vehicle (2% HPMC E-50, 0.5% Tween-80 in 50 mM Sodium Citrate Buffer, pH 4.0), 5 mg/kg osimertinib q.d., 50 mg/kg VS-4718 b.i.d., or combinations of osimertinib with VS-4718. For TEAD inhibitor VT108 models, once the tumors reached an average size of ~200 mm^3^, mice were randomly assigned to receive treatment with vehicle (0.5% HPMC + 0.1% Tween80 mixture1 and 5% DMSO + 10% solutol+85% D5W mixture2 are administrated with a 30-minute interval between them), 2.5 mg/kg osimertinib (in 0.5% HPMC + 0.1% Tween80 mixture1) q.d., 30 mg/kg VT108 (in 5% DMSO + 10% solutol+85% D5W mixture2) q.d., or combinations of osimertinib with VT108. No substantial toxicity was observed in mice treated with either combination regimen incorporating FAK or TEAD inhibitors by assessment of body weight (Supplementary Fig. 8c) and general animal well-being. EGFR-mutant TH021 and ALK fusion-positive LG0812 PDX, tumors were propagated into 6- to 8-wk-old female SCID mice. Once the tumors grew to an average size of ~400 mm^3^, mice were randomized and treated with vehicle, 10 mg/kg osimertinib q.d. (TH021) or 6 mg/kg alectinib q.d. (LG0812) following by immunohistochemistry (IHC) for nuclear YAP staining. For the EGFR-mutant TH021, LU-01-1291 and LU1868 PDX models, combinatorial treatment effects were assessed upon treatment with vehicle, 2.5 mg/kg osimertinib q.d., 30 mg/kg VT108 q.d. as well as combinations of osimertinib with VT108. Tumor volume was assessed regularly. At the treatment endpoint, tumors were halved and harvested in ice-cold PBS.

### Humanized mouse model

Humanized xenograft models were kindly established by the laboratory of Jack Roth at MD Anderson Cancer Center as described previously^41^. All animal use was conducted in accordance with the guidelines of the Animal Care and Use Committee of MD Anderson Cancer Center. In brief, female 3-to-4-week-old NOD. Cg-Prkdcscid Il2rgtm1Wjl/SzJ (NSG) mice, which are suitable for the engraftment of human hematopoietic cells, were housed in microisolator cages under specific pathogen-free conditions in a dedicated humanized mice room in the animal facility at The University of Texas MD Anderson Cancer Center. Mice were given autoclaved acidified water and fed a special diet (Uniprim diet). Human umbilical cord blood units were obtained from MD Anderson Cord Blood Bank under an IRB-approved protocol. Fresh cord blood units were delivered within 24 h of harvest and were HLA typed immediately at MD Anderson HLA-typing core facility. Cord blood was diluted to a ratio of 1:3 with phosphate-buffered saline, and mononuclear cells were isolated by using density-gradient centrifugation on Ficoll medium. CD34+ HSPCs were isolated using a direct CD34+ MicroBead kit (Miltenyi Biotec). NSG mice were irradiated with 200 cGy using a 137Cs gamma irradiator. Over 90% pure freshly isolated CD34+ HSPCs were injected intravenously, 24 h after irradiation, at a density of 1 to 2 × 105 CD34+ cells/ mouse. All Hu-NSG mice were verified for humanization before tumor implantation. For PC9-parental, PC9-WT (YAP overexpression), PC9-5SA (hyperactive YAP) cell lines, 5-7 × 106 cells were injected subcutaneously 8-weeks post humanization of mice. Another 3-4 weeks post tumor cells implantation in humanized mice and when tumor sizes reached 200mm3, animals were randomized into treatment and no-treatment groups based on tumor size and donor HLA type. Five mice per group from multiple umbilical cord blood donors were used. Mice were treated with vehicle or osimertinib (5 mg/kg) orally 5 days a week for consecutive 3 weeks. For immune analysis, erythrocytes in the peripheral blood were lysed with ACK lysis buffer (Fisher Scientific). Single-cell suspensions were prepared. Several 10-color flow cytometry panels were used for immune profiling of both innate and adaptive immune populations in humanized mice and for evaluating immune response after treatment. All samples were run on Attune NxT flow cytometer (Thermo fisher), and data were analyzed by Flow Jo and Kaluza software packages.

### Immunohistochemistry

Formalin-fixed paraffin-embedded (FFPE) tumor blocks were cut at 4-micron thickness and mounted as sections on positively charged histology slides. Immunohistochemistry staining was performed as described previously^69^. In brief, slides were deparaffinized in xylene, rehydrated and epitope retrieval was induced in a histology pressure cooker using pH 6.1 citrate buffer (Dako Denmark A/S, S2369). After endogenous peroxidase and protein block, slides were incubated with primary antibody solution overnight at 4 °C. Then slides were incubated with secondary antibody for 30 minutes (EnVision Dual Link Labeled Polymer HRP, Agilent K4065), stained using 3,3-DAB, and counterstained with hematoxylin. Slides were dehydrated and mounted before digitization using an Aperio AT2 Slide Scanner (Leica Biosystems) at a 20X objective. Quantification of nuclear YAP levels was performed via the Aperio Image Scope digital pathology software using the nuclear quantification algorithm.

### YAP signature in scRNA-seq data of patient samples

Single-cell sequencing data were derived from previously published work^5^ and filtered to limit analyzes to malignant lung epithelial cells only (*N* cells: TN = 621, RD = 484, PD = 138). Differentially expressed gene sets for YAP activation (YAP-5SA-UP), targeted therapy-derived DTPs models (PC9⋂H3122⋂H358), and patient treatment timepoint (TN, RD, PD) were compared to the patient gene expression dataset via permutation analysis (R package, GSALightning, v.1.1.7). Subject classes were assigned to every single cell based on the corresponding time point, where RD = “RD” and TN or PD = “nonRD.” Each gene was tested for significance with unpaired t-tests and gene set statistics were calculated from the mean of respective gene constituents. Multiple testing correction was done via Benjamini-Hochberg. The intersection of significantly upregulated genes (adjusted *p*-value < 0.05) from the YAP-5SA_UP gene set, significantly upregulated genes across DTPs models, and the RD treatment timepoint were then used to define the YAP gene signature. Other signatures (i.e., cell cycle, FAK) were determined from relevant literature^5,30^. Signature expression for single-cell data was processed and plotted in R with ggplot2 (v.3.3.3) and Seurat (v.3.2.2). Source code for sequencing analysis from scRNA seq data of patient specimens is available here: https://github.com/BivonaUCSF/YAP.git (10.5281/zenodo.10632418)^64^.

### LINCS L1000 concordance score

The NIH LINCS L1000 database^43^ contains gene expression data from cultured human cells treated with small molecule and genetic perturbagens. Level 4 data was sourced from the Gene Expression Omnibus Series GSE70138. Expression data was restricted to small molecule perturbagens and intersected with the residual disease signature (*N* = 83 genes). Using a previously published computational pipeline^70,71^, a score for each signature-drug pair was determined using a non-parametric rank-based method that is similar to the Kolmogorov–Smirnov test statistic, where negative scores indicate genes in the ranked drug profile are oppositely regulated in the ranked disease signature. *P*-values for drug-gene expression profiles were determined by comparing their scores to a distribution of random scores and adjusted with the false discovery rate (FDR; Benjamini-Hochberg, α = 0.05) method. Metadata and identifiers associated with each perturbagen were sourced and validated from the iLINCS suite^43^. For the upregulated residual disease signature, drug-gene expression profiles were chosen that produced the greatest significant negative score.

### Statistical analysis

Quantitative data are presented as mean +/− standard deviation (S.D.). Statistical tests were performed using GraphPad Prism 8.4.2. Two-sided Student’s *t*-tests were used for comparisons of the means of data between two groups unless otherwise specified. For comparisons among multiple independent groups, a one-way ANOVA test was used. For animal studies, animals were randomized before treatments, and all animals treated were included in the analyzes.

### Reporting summary

Further information on research design is available in the Nature Portfolio Reporting Summary linked to this article.

### Supplementary information

Supplementary Information

Reporting Summary

Description of Additional Supplementary Files

Supplementary Data 1-8

### Source data

Source Data

## Data Availability

The datasets generated during and/or analyzed during the current study have been deposited as an NCBI Bioproject under accession number PRJNA766057. The data used for analyzes of patient specimens, which is referenced in the text and figure legends^5^ is available as an NCBI Bioproject under accession number PRJNA591860. We also used publicly available datasets in this study, which are referenced in the text and figure legends^43^ and can be found in the GEO database under accession code GSE70138. The remaining data are available within the Article, Supplementary Information, or Source Data file. Biological material (e.g. cell lines, plasmids) generated in this study is available by request from the corresponding author. Additional reagents can be made available upon reasonable request. Source data are provided as a Source Data file. Source data are provided with this paper.

## **Code availability**

The codes generated during the current study are available in the Supplementary Information or on Github: https://github.com/fhaderk/NucYAP.git (10.5281/zenodo.10614956)^61^ and https://github.com/BivonaUCSF/YAP.git (10.5281/zenodo.10632418)^64^.
