# Supplementary material for: Focal adhesion kinase-YAP signaling axis drives drug-tolerant persister cells and residual disease in lung cancer
Source: Nat Commun. 2024 May 3;15:3741. doi: 10.1038/s41467-024-47423-0 (PMC11068778; doi:10.1038/s41467-024-47423-0)
Supplement: Supplementary file 1 — Supplementary Information [file 41467_2024_47423_MOESM1_ESM.pdf]

Title: Focal adhesion kinase-YAP signaling axis drives drug-tolerant persister cells and residual disease in lung cancer

Supplementary Figures

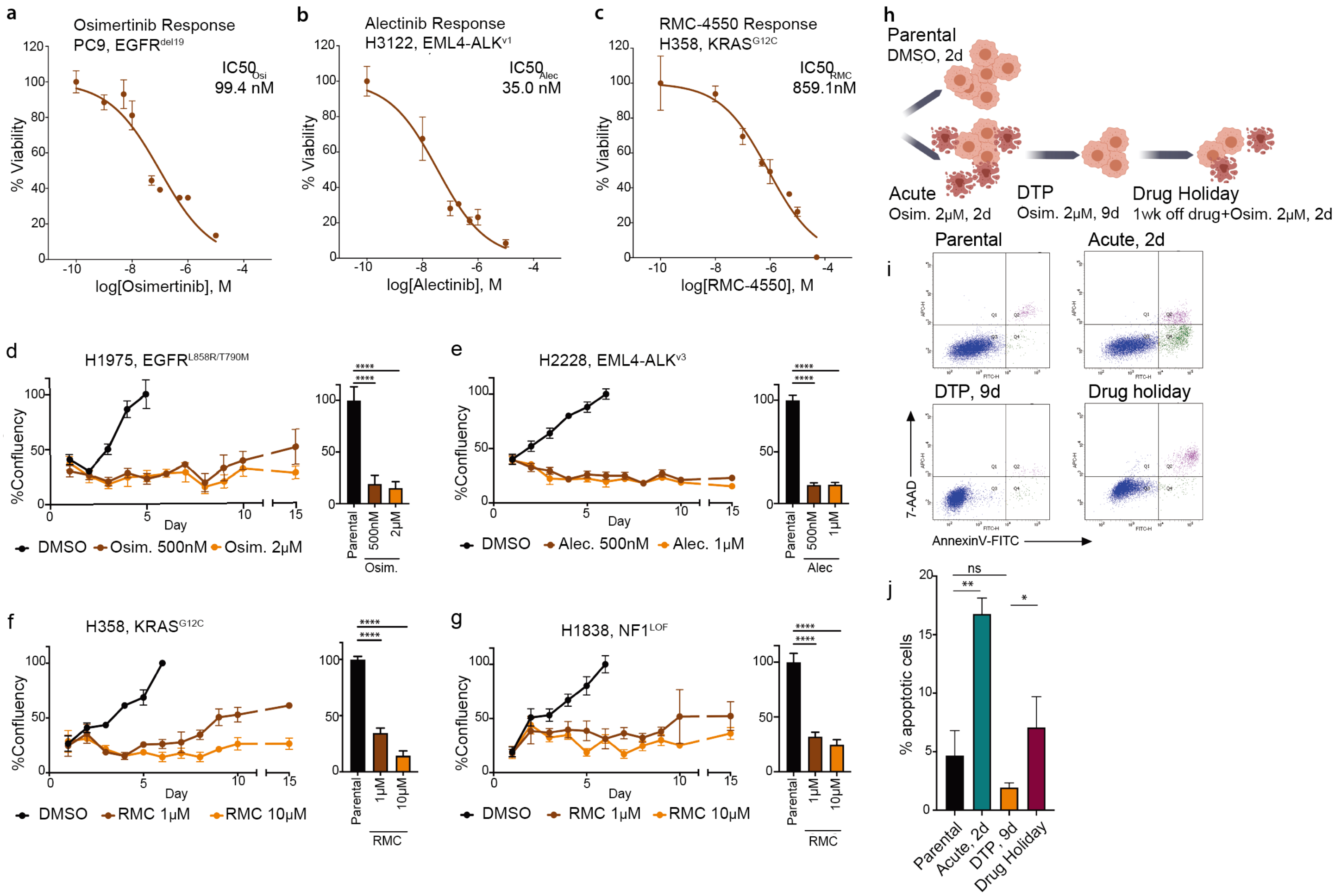

Supplementary Fig. 1. Characterization of drug-tolerant persister cells (DTPs).

(**a-c**) Drug response curve to targeted inhibitors across EGFR-mutant PC9 cells treated with osimertinib, ALK fusion-positive H3122 cells treated with alectinib, and KRAS-mutant H358 cells treated with RMC-4550. IC50, half maximal inhibitory concentration. (**d-g**) High-content microscopy screen monitoring relative cell numbers in cells treated with targeted inhibitors. (**d-g, left**) The graph shows the confluency of (**d**) EGFR-mutant H1975 cells, (**e**) ALK-fusion positive H2228 cells, (**f**) KRAS-mutant H358 cells, and (**g**) NF1-mutant H1838 cells treated with different dosage of osimertinib, alectinib, and RMC-4550, compared to 0.1 % DMSO control. *n* = 6 per data point.

(**d-g, right**) The bar graph compares the total cell counts between the parental cells and DTPs at day 8. (**h**) Schematic representation of apoptosis in parental, acute, drug-tolerant, and drug-free conditions. Schematic diagram was created with BioRender.com. (**i-j**) Monitoring of apoptosis by Annexin-V / 7-AAD staining in EGFR-mutant PC9 cells, including untreated parental cells (0.1 % DMSO), acutely treated cells (2-day osimertinib 2 μM), DTP cells (9-day osimertinib 2 μM), and DTP cells that were cultured off-drug for 7 days prior to re-treatment with 2 μM osimertinib for 2 days. $n = 6$ per data point. Statistical evaluation by unpaired t-test. Parental vs Acute, ** $p = 0.0012$; parental vs drug-tolerant persisters, ns, $p = 0.0950$; parental vs drug holiday, * $p = 0.0288$.

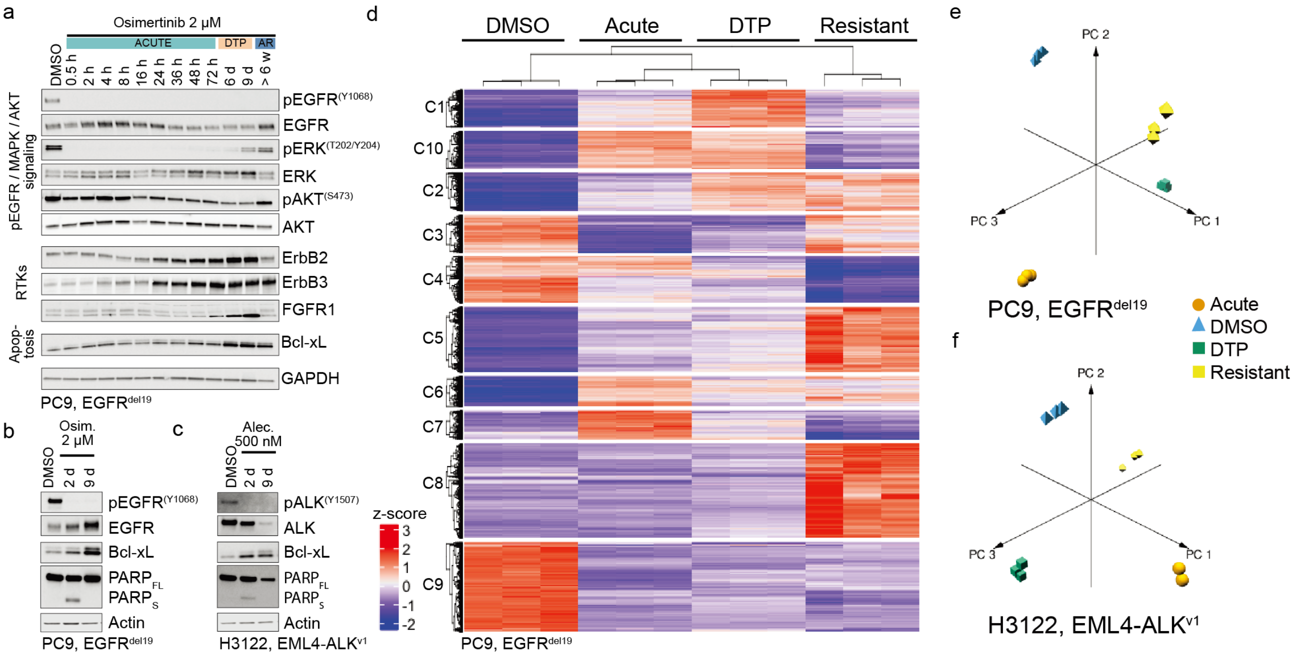

Supplementary Fig. 2. Signaling and transcriptional changes in DTPs.

(**a**) Signaling changes during DTP development in EGFR-mutant PC9 cells treated with 2 µM osimertinib. Specifically, it delved on osimertinib-mediated suppression of EGFR-ERK signaling, and evaluate the changes in protein expression levels of receptor tyrosine kinases ErbB2, ErbB3, FGFR1, and FGFR2, alongside the anti-apoptotic Bcl-xL. (**b-c**) Monitoring on-target signaling suppression by targeted inhibitors in acute treatment (2d) and in DTPs (9d). This was assessed through the examination of phosphor-EGFR at Tyr1068 in osimertinib-treated PC9 cells and phosphor-ALK at Tyr1507 in alectinib-treated H3122 cells. Alterations in apoptosis during drug-tolerant cell generation with elevated levels of apoptosis-associated cleaved PARP ($PARP_S$) in acutely treated cells (2d), while DTPs (9d) showed an absence of cleaved PARP and an increase of anti-apoptotic protein Bcl-xL. (**d-f**) Changes in RNA expression across 0.1 % DMSO treated parental cells (DMSO), acutely treated cells (Acute), drug-tolerant persisters (DTP), and acquired resistant cells (Resistant). (**d**) Heatmap analysis of differentially expressed genes in EGFR-mutant PC9 cells and derived acquired resistant PC9-AR cells treated with 2 µM osimertinib. (**e-f**) Principal component analysis across treatment states for EGFR-mutant PC9 cells treated with osimertinib and ALK fusion-positive H3122 cells treated with alectinib.

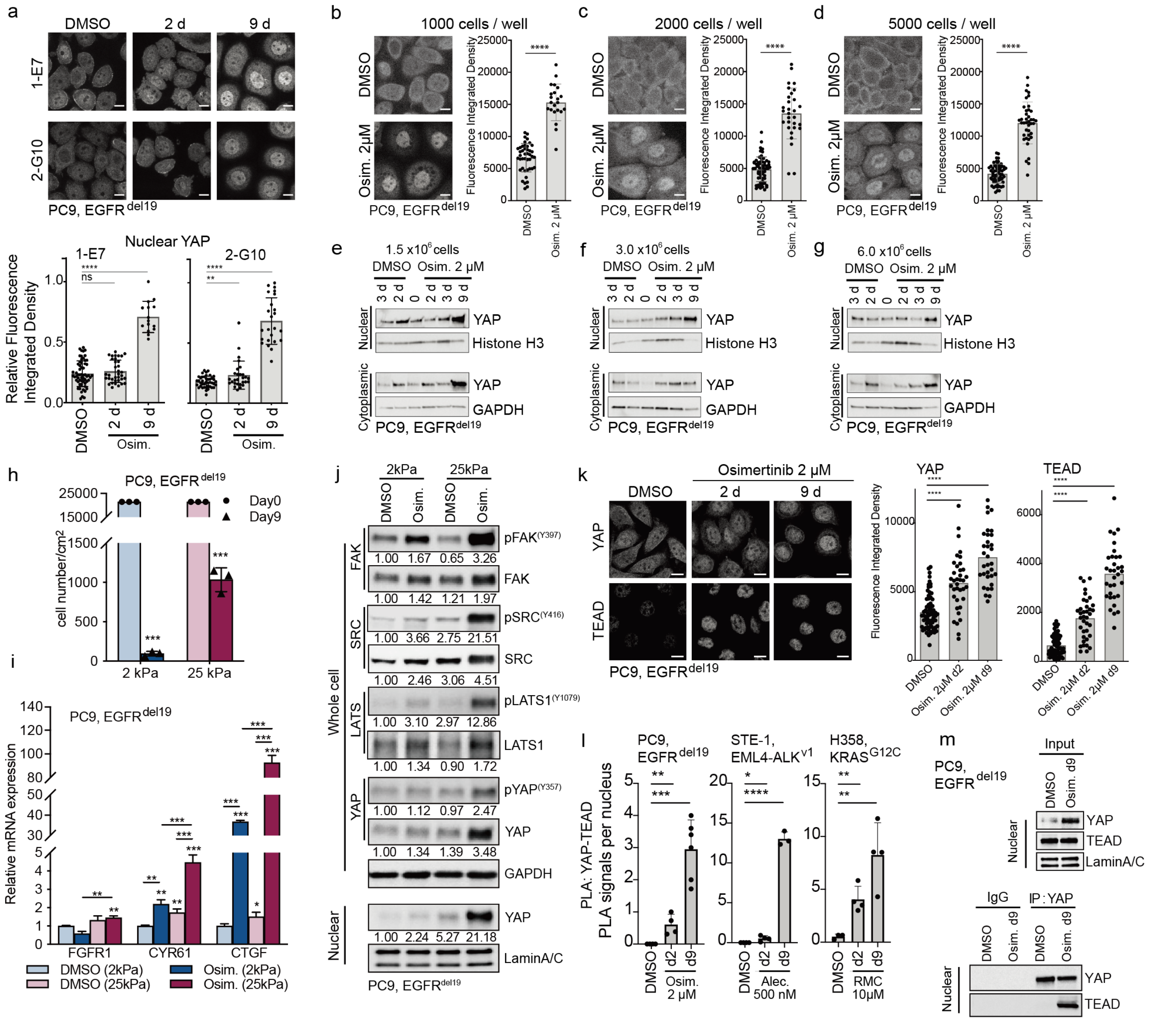

Supplementary Fig. 3. YAP is engaged in DTPs under different conditions.

(**a**) Generation of isogenic, endogenously mNeonGreen (mNG)-tagged YAP PC9 cell lines 1-E7 and 2-G10 and monitoring of changes in YAP nuclear levels by confocal microscopy. (**a, bottom**) Quantification of fluorescence integrated density per nuclei in 1-E7 and 2-G10 confirmed significant increase in YAP nuclear levels in DTPs. (**b-d**) Analysis of YAP nuclear levels upon osimertinib treatment in PC9 cells seeded at different densities in 96-well format. Analysis was conducted using confocal microscopy on day 5 post-seeding. (**e-g**) Analysis of YAP

nuclear and cytoplasmatic levels upon osimertinib treatment in PC9 cells seeded at different densities in 150 mm dishes. Analysis at indicated timepoints after treatment initiation was performed using immunoblot. (**h**) The osimertinib-tolerant phenotype of PC9 with 25kPa stiffness was higher than that of PC9 with 2kPa stiffness. Followed by treating PC9 cells with 100nM osimertinib for 0 or 9 days, the total number of cells was calculated by hemocytometer and averaged by the surface area of the plate (cm^2). (**i**) Osimertinib induced higher YAP target gene expression with different stiffness. In the condition with 25kPa stiffness matrix, YAP target genes had a more significant increase than at 2kPa stiffness. (**j**) Osimertinib induced phosphorylation and activation of FAK, SRC and YAP with a 25kPa stiffness matrix. Nuclear YAP protein expression was also elevated with osimertinib treatment with 25kPa stiffness compared to DMSO control. (**k**) Nuclear localization of YAP and TEAD were enriched in PC9 osimertinib DTPs, as analyzed by confocal microscopy. Representative images (left), scale bar: 10 µm. Quantification of relative integrated density for nuclear levels was performed by automated analysis quantifying the intensity for the protein of interest per nuclei (right). (**l**) The panTEAD-YAP proximity ligation assay (PLA) was conducted in PC9, STE-1, and H358 cells. PLA positive signals per nucleus were quantified as mean value along with standard deviation. n = 3-6 per condition. (**m**) YAP co-immunoprecipitation (Co-IP) and analysis for concurrent pulldown of TEAD in nuclear fractions of PC9 osimertinib DTPs. Statistical evaluation by unpaired t-test with ns, $p > 0.05$; * $p \leq 0.05$; ** $p \leq 0.01$; *** $p \leq 0.001$; **** $p < 0.0001$.

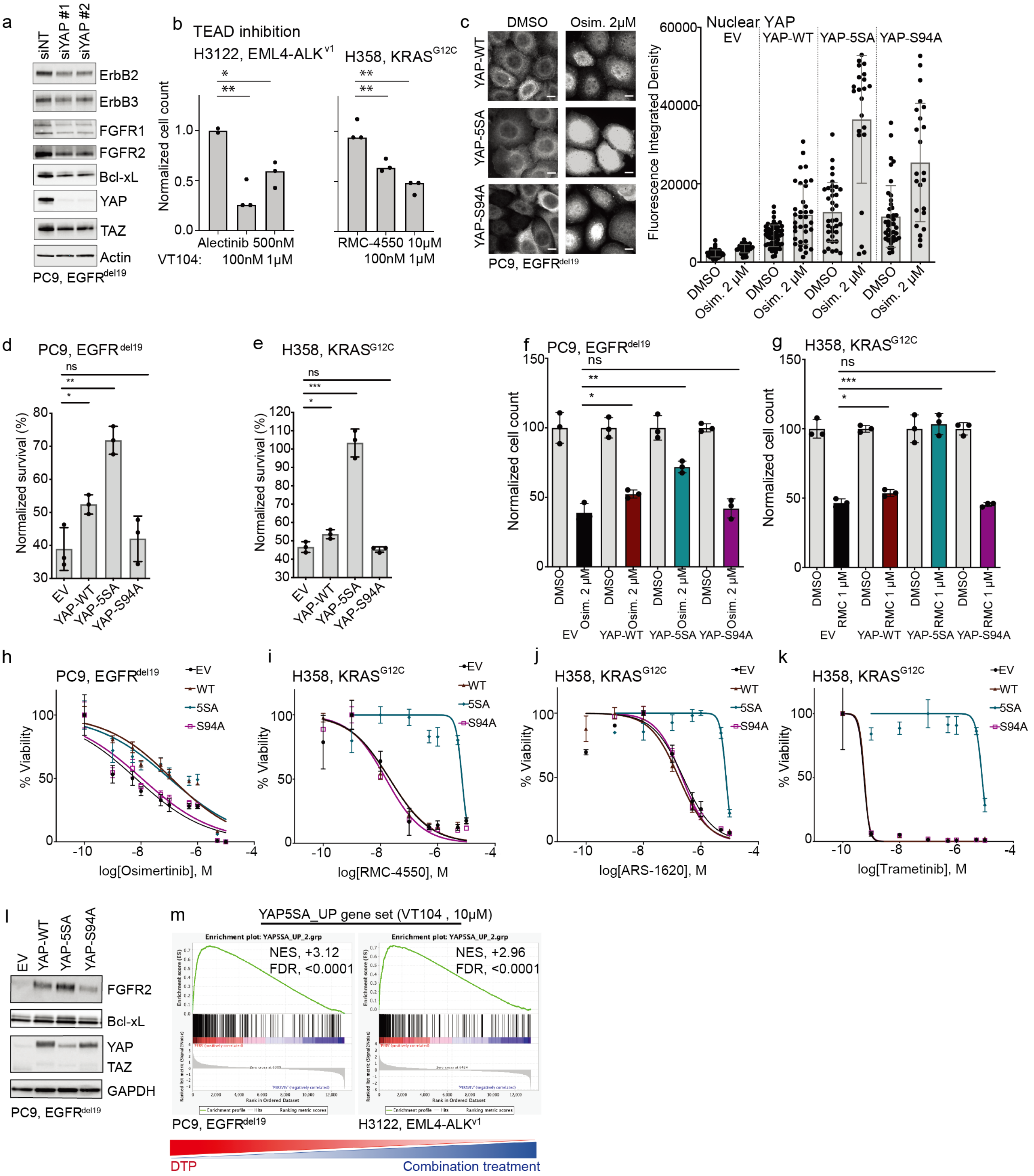
a
siNT
siYAP #1
siYAP #2
ErbB2
ErbB3
FGFR1
FGFR2
Bcl-xL
YAP
TAZ
Actin
PC9, EGFRdel19
b
TEAD inhibition
H3122, EML4-ALKv1
H358, KRASG12C
Normalized cell count
Alectinib 500nM
RMC-4550 10μM
VT104: 100nM 1μM
100nM 1μM
c
DMSO
Osim. 2μM
YAP-WT
YAP-5SA
YAP-S94A
PC9, EGFRdel19
Nuclear YAP
EV
YAP-WT
YAP-5SA
YAP-S94A
Fluorescence Integrated Density
DMSO
Osim. 2 μM
d
PC9, EGFRdel19
Normalized survival (%)
EV
YAP-WT
YAP-5SA
YAP-S94A
e
H358, KRASG12C
f
PC9, EGFRdel19
Normalized cell count
g
H358, KRASG12C
RMC 1 μM
h
PC9, EGFRdel19
% Viability
log[Osimertinib], M
EV
WT
5SA
S94A
i
H358, KRASG12C
log[RMC-4550], M
j
H358, KRASG12C
log[ARS-1620], M
k
H358, KRASG12C
log[Trametinib], M
l
EV
YAP-WT
YAP-5SA
YAP-S94A
FGFR2
Bcl-xL
YAP
TAZ
GAPDH
PC9, EGFRdel19
m
YAP5SA_UP gene set (VT104 , 10μM)
NES, +3.12
FDR, <0.0001
NES, +2.96
FDR, <0.0001
PC9, EGFRdel19
H3122, EML4-ALKv1
DTP
Combination treatment

Supplementary Fig. 4. YAP activation can promote drug tolerance.

(**a**) Changes in protein expression of receptor tyrosine kinases ErbB2, ErbB3, FGFR1 and FGFR2 and anti-apoptotic protein Bcl-xL upon siRNA-mediated YAP knockdown in PC9 osimertinib DTPs. (**b**) Normalized cell number for H3122 alectinib DTPs and H358 RMC-4550 DTPs upon combined treatment with TEAD inhibitor VT104. Statistical evaluation by unpaired t-test with * $p \leq 0.05$ and ** $p \leq 0.01$. (**c-g**) YAP nuclear localization (**c**) and relative survival (**d-g**) upon treatment with targeted inhibitors for 48 hours in EGFR-mutant PC9 cells and KRAS-mutant H358 cells overexpressing YAP-WT, hyperactive YAP-5SA, and functionally inactive YAP-S94A, respectively. For (**d-g**), statistical evaluation by unpaired t-test. PC9: empty vector (EV) vs YAP-WT, * $p = 0.0301$; EV vs YAP-5SA, ** $p = 0.0018$; EV vs YAP-S94A, ns, $p = 0.5990$. H358: EV vs YAP-WT, * $p = 0.0324$; EV vs YAP-5SA, *** $p = 0.0003$; EV vs YAP-S94A, ns, $p = 0.5278$. (**h-k**) Treatment response to targeted inhibitors upon overexpression of YAP-WT, hyperactive YAP-5SA, and functionally inactive YAP-S94A, respectively; as shown for the response to osimertinib in (**h**) PC9 cells, as well as to (**i**) RMC-4550, (**j**) ARS-1620, and (**k**) Trametinib in H358 cells. (**l**) Expression changes in YAP target genes Bcl-xL and FGFR2 upon expression of YAP-WT, hyperactive YAP-5SA, and functionally inactive YAP-S94A, respectively, in EGFR-mutant PC9 cells. (**m**) Gene set enrichment analysis (GSEA) was performed using the YAP-5SA_UP gene set (Supplementary Data 3) on RNAseq expression data from PC9 osimertinib DTPs and H3122 alectinib DTPs, both treated with 10 μM VT104 for 24 h. *NES*, Nominal Enrichment Score; *FDR*, False Discovery Rate.

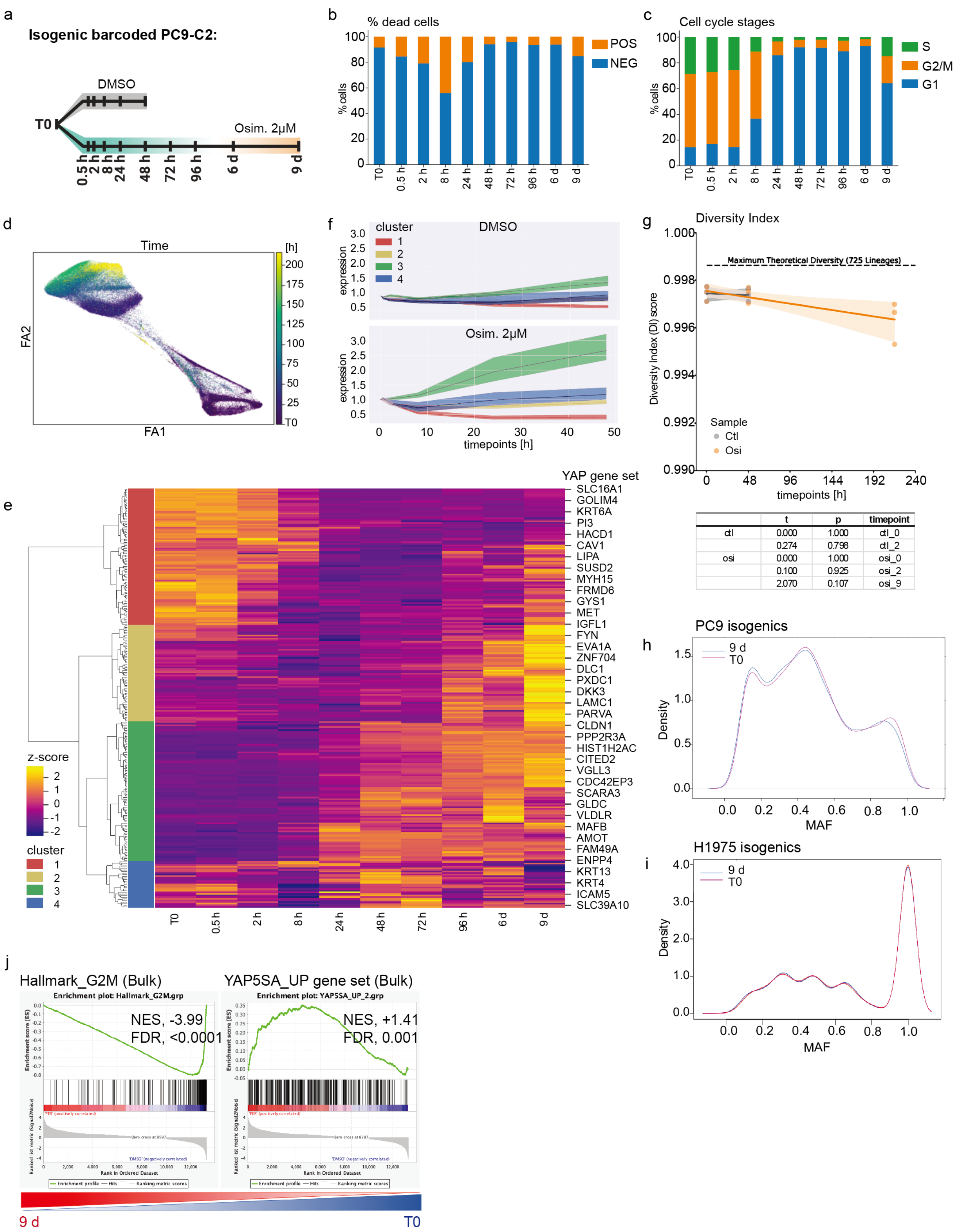

	t	p	timepoint
ctl	0.000	1.000	ctl_0
	0.274	0.798	ctl_2
osi	0.000	1.000	osi_0
	0.100	0.925	osi_2
	2.070	0.107	osi_9

Supplementary Fig. 5. Single cell RNA sequencing trajectories during treatment of NSCLC cells.

(**a**) Experimental outline indicating treatment conditions and time points for single cell RNA (scRNA) sequencing trajectory study. Schematic created with BioRender.com. (**b-d**) Expression changes upon treatment with osimertinib, showing the evaluation of (**b**) the percentage (%) of dead cells and (**c**) the distribution of cell cycle stages, as well as (**d**) the transcriptional development over the time of treatment with a noticeable shift in expression from early (t0 to 8h) to later time pointes (> 24h). (**e**) Heatmap of YAP-associated transcriptional targets (YAP-5SA_UP gene set) along trajectory in osimertinib-treated conditions, with unsupervised hierarchical clustering according to differences in expression patterns identified at single cell level. (**f**) Relative expression for YAP gene set clusters upon treatment with 0.1% DMSO control or 2 μM osimertinib. The YAP genes in cluster 3 (green) show a time-dependent modulation after osimertinib treatment, indicating selective enrichment of YAP cluster 3 gene expression. (**g**) Diversity index based on the relative enrichment or decrease of genetic barcodes in DMSO and osimertinib trajectories with statistical evaluation demonstrating no significant selection of genetically labeled cell subsets. (**h-i**) Density blot of mutant allele frequencies (MAF) identified by whole exome sequencing and comparing cells prior to treatment (t0) versus at day 9 of osimertinib treatment, showing identical profiles in both conditions and no shift in MAF density under treatment. (**j**) Independent validation of transcriptional changes by bulk RNA sequencing and gene set enrichment analysis for Hallmark_G2M and YAP-5SA_UP gene sets across isogenic EGFR-mutant cell lines, with combined sequencing analysis of isogenic PC9 and H1975 cells (each $n = 2$) and comparing cells prior to treatment (t0) versus at day 9 of osimertinib treatment. *NES*, Nominal Enrichment Score; *FDR*, False Discovery Rate.

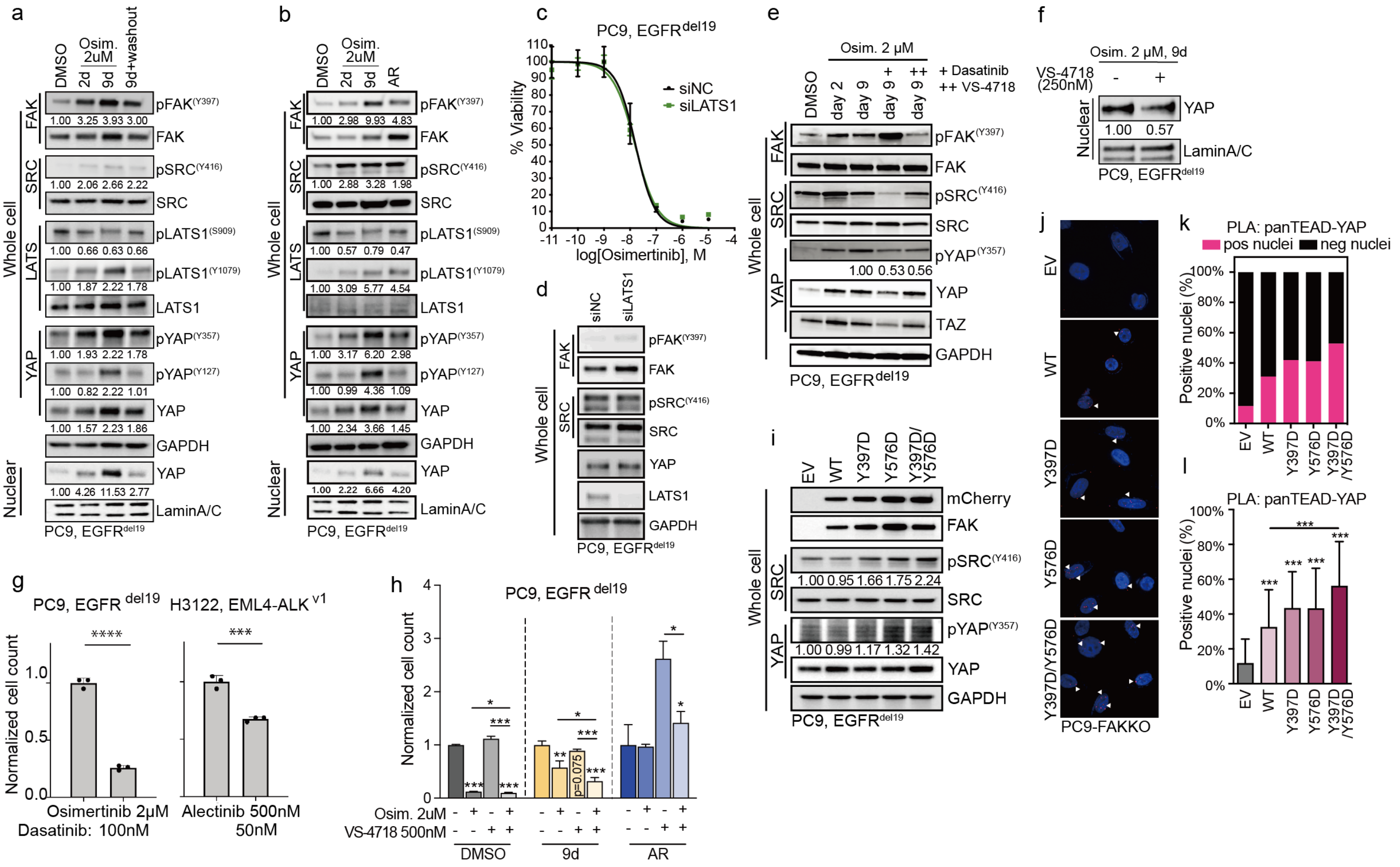

Supplementary Fig. 6. FAK signaling promotes YAP signaling activation to support DTPs.

(**a-b**) Phosphorylation and activation changes of SRC were observed specifically in PC9 osimertinib-DTP cells. Upon treatment with targeted inhibitors, activated FAK Tyr397 and YAP Y357 were also upregulated in EGFR-mutant PC9-DTP cells in contrast to (a) washout and (b) acquired resistance (AR) conditions. (**c**) Knockdown of LATS1 did not alter Osimertinib sensitivity and drug tolerance in PC9 cells. Cells were treated with the indicated concentration of osimertinib for 8 days before performing CellTiterGlo analysis for the viability assay. (**d**) FAK and SRC signaling was not regulated by LATS1 in PC9 cells. (**e**) Signaling changes during DTPs development in PC9 cells treated with 2 μM osimertinib. Where indicated, short-term treatment of 50 nM Dasatinib (+) or 1 μM VS-4718 (++) has been added for 24 hours prior to harvest. Signaling analysis focuses on combinatorial treatment-mediated suppression of SRC-FAK signaling and the changes of YAP at Tyr357 activating phosphorylation. (**f**) Inhibition of FAK by the FAK inhibitor VS4718 (250nM) decreased nuclear YAP expression

levels in PC9-DTP cells. (**g**) Normalized PC9 and H3122 cell numbers upon combined treatment with targeted therapy and dasatinib during DTP generation. Statistical evaluation by unpaired t-test. *** $p = 0.0002$; **** $p < 0.0001$. (**h**) Effects of FAK inhibitor (VS-4718) in combination with osimertinib on PC9 DTPs but not in 0.1 % DMSO control and acquired resistant PC9. (**i**) Overexpression of hyperactive FAK induced phosphorylation and activation of SRC at Tyr416 and YAP at Tyr357. Hyperactive FAK mutants were derived via phospho-mimetic mutant generation by site-direct mutagenesis at Tyr397 and Tyr576. (**j**) Hyperactive-FAK induced more nuclear panTEAD and YAP interaction (white arrowhead) in PC9 FAKKO cells. (**k-l**) Quantification of percentage WT- or Hyperactive-FAK cells with positive PLA signal in the nuclear (3 or more), 400 or more cells per condition.

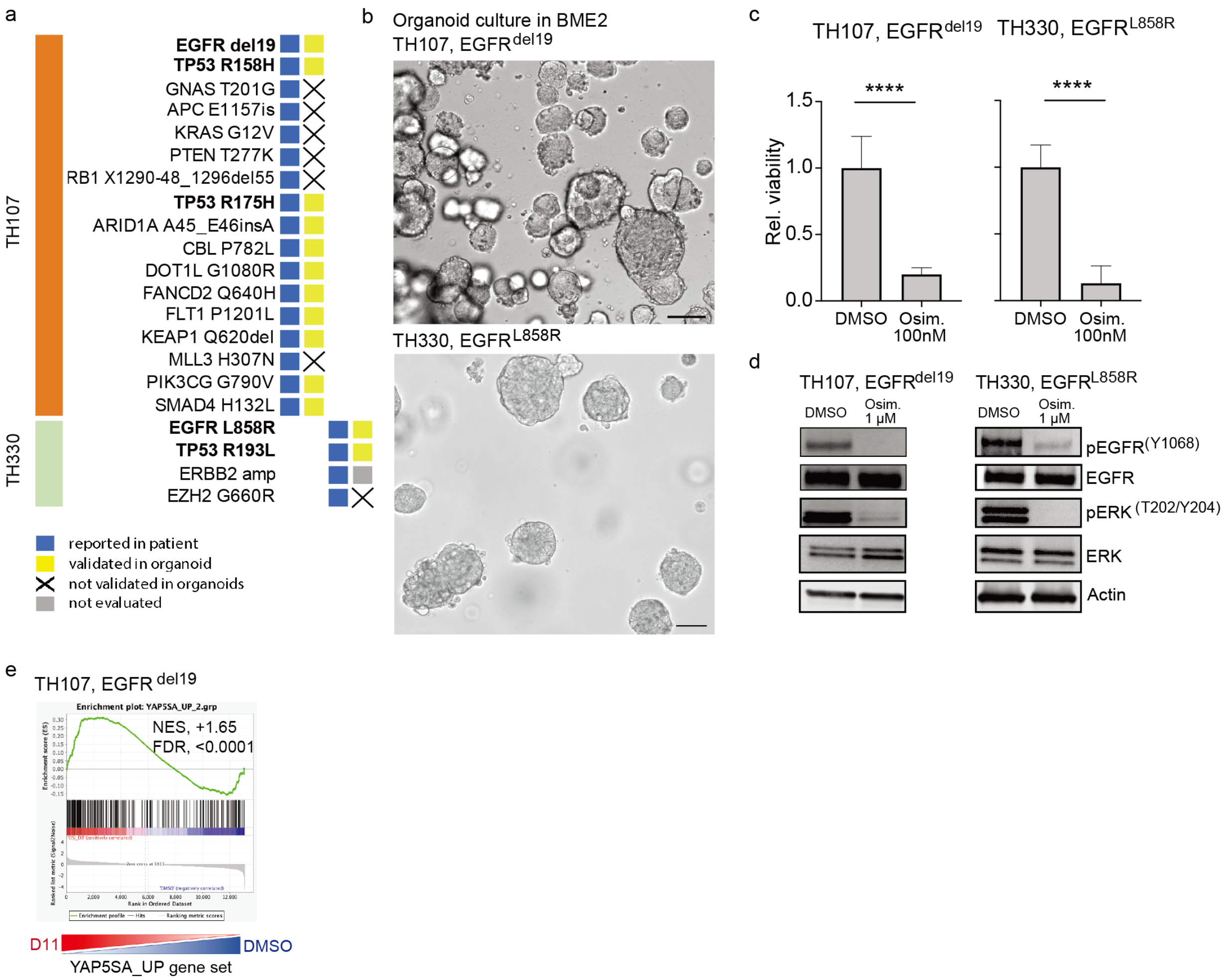

Supplementary Fig. 7. Treatment response in PDO models.

(**a**) Mutation profile of patient NSCLC specimen and corresponding patient-derived organoid (PDO) models, with indications for the validation status of clinically reported tumor mutation. (**b**) Representative examples of organoid cultures for EGFR-mutant PDOs TH107 ($EGFR^{del19}$) and TH330 ($EGFR^{L858R}$); scale bar: 100 µm. (**c**) Sensitivity of EGFR-mutant PDOs to 100 nM osimertinib treatment. (**d**) Suppression of EGFR-ERK signaling upon 1 µM osimertinib treatment in EGFR-mutant PDOs. (**e**) Gene set enrichment analysis for the YAP-5SA_UP gene set (Supplementary Data 3) using RNAseq expression data of the EGFR-mutant TH107 PDO model, comparing untreated DMSO control (DMSO) versus osimertinib persisters (D11). *NES*, Nominal Enrichment Score; *FDR*, False Discovery Rate.

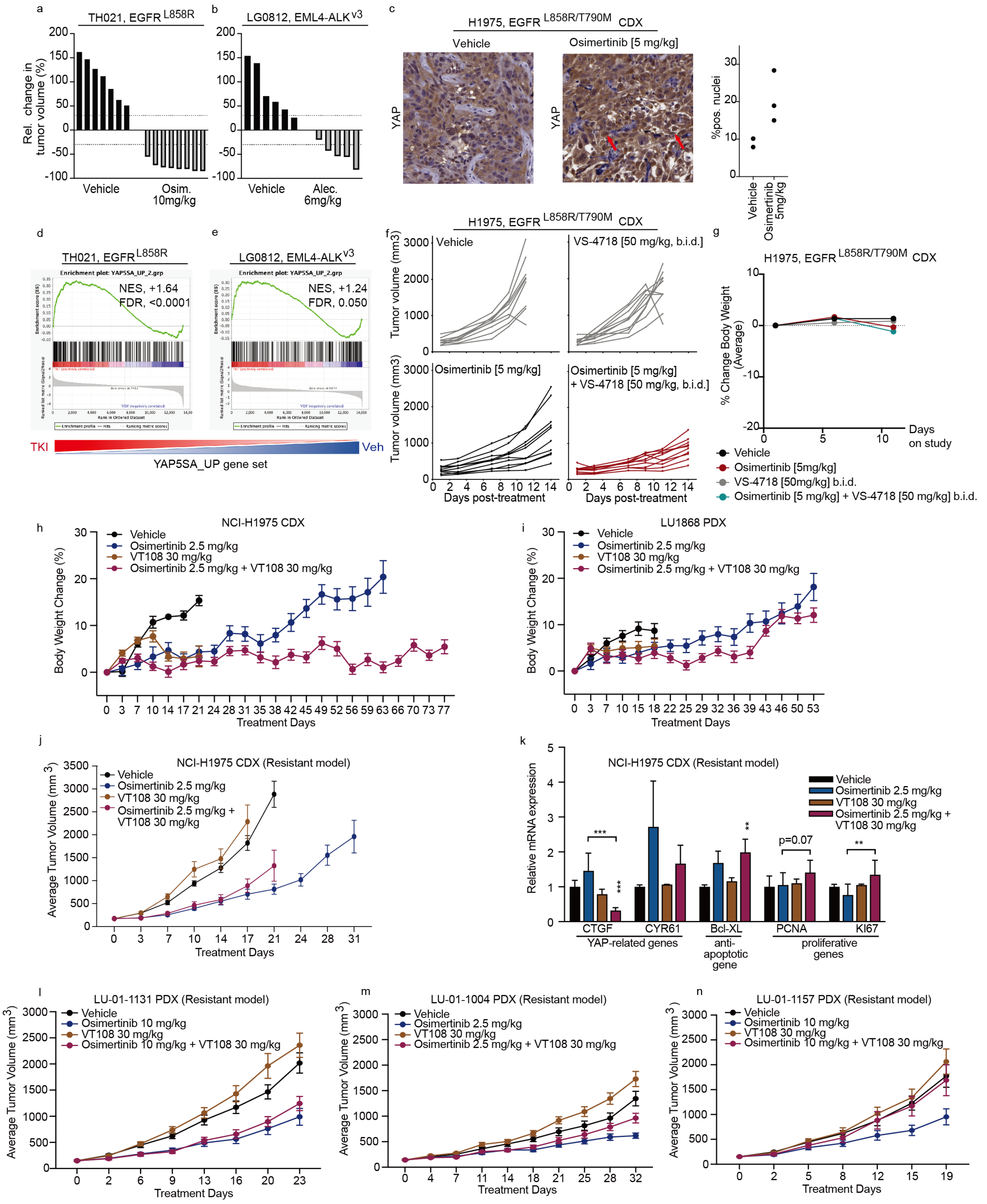
a
TH021, EGFR L858R
Rel. change in tumor volume (%)
Vehicle
Osim. 10mg/kg
b
LG0812, EML4-ALK v3
Vehicle
Alec. 6mg/kg
c
H1975, EGFR L858R/T790M CDX
Vehicle
Osimertinib [5 mg/kg]
YAP
%pos. nuclei
Vehicle
Osimertinib 5mg/kg
d
TH021, EGFR L858R
NES, +1.64
FDR, <0.0001
e
LG0812, EML4-ALK v3
NES, +1.24
FDR, 0.050
TKI
Veh
YAP5SA_UP gene set
f
H1975, EGFR L858R/T790M CDX
Tumor volume (mm3)
Vehicle
VS-4718 [50 mg/kg, b.i.d.]
Osimertinib [5 mg/kg]
Osimertinib [5 mg/kg] + VS-4718 [50 mg/kg, b.i.d.]
Days post-treatment
g
H1975, EGFR L858R/T790M CDX
% Change Body Weight (Average)
Days on study
Vehicle
Osimertinib [5mg/kg]
VS-4718 [50mg/kg] b.i.d.
Osimertinib [5 mg/kg] + VS-4718 [50 mg/kg] b.i.d.
h
NCI-H1975 CDX
Body Weight Change (%)
Vehicle
Osimertinib 2.5 mg/kg
VT108 30 mg/kg
Osimertinib 2.5 mg/kg + VT108 30 mg/kg
Treatment Days
i
LU1868 PDX
Body Weight Change (%)
Treatment Days
j
NCI-H1975 CDX (Resistant model)
Average Tumor Volume (mm 3)
Treatment Days
k
NCI-H1975 CDX (Resistant model)
Relative mRNA expression
p=0.07
CTGF
CYR61
Bcl-XL
PCNA
KI67
YAP-related genes
anti-apoptotic gene
proliferative genes
l
LU-01-1131 PDX (Resistant model)
Osimertinib 10 mg/kg
Osimertinib 10 mg/kg + VT108 30 mg/kg
m
LU-01-1004 PDX (Resistant model)
n
LU-01-1157 PDX (Resistant model)
Average Tumor Volume (mm3)
Treatment Days

3

Supplementary Fig. 8. Combinatorial treatment studies in EGFR-mutant CDX and PDX models. (**a-b**) Relative change in tumor volume for EGFR-mutant PDX TH021 (EGFR^{L858R}) treated with 10 mg/kg osimertinib for 7 days (**a**) and for ALK fusion-positive PDX LG0812 (EML4-ALK^{v3}) treated with 6 mg/kg alectinib for 17 days (**b**), compared to vehicle control. (**c**) Immunohistochemistry staining for YAP in H1975 xenograft tumor specimens from animals treated with vehicle or 5 mg/kg osimertinib. Quantification of nuclear levels (% nuclear) by automated image analysis. Arrows indicate cluster of YAP positive tumor cell nucleus. (**d-e**) Gene set enrichment analysis for the YAP-5SA_UP gene set (Supplementary Data 3) using RNAseq expression data of the EGFR-mutant TH021 PDX model (**d**) and ALK fusion-positive LG0812 PDX model (**e**), comparing vehicle control (VEH) versus treatment group (TKI). *NES*, Nominal Enrichment Score; *FDR*, False Discovery Rate. (**f**) Relative tumor volume development for individual H1975 xenograft treatment study across vehicle, 5 mg/kg osimertinib, 50 mg/kg FAK inhibitor VS-4718, and combinatorial 5 mg/kg osimertinib + 50 mg/kg FAK inhibitor VS-4718 treatment groups. (**g**) Percent change in body weight (BW) for the H1975 xenograft treatment study with FAK inhibitor VS-4718. (**h-i**) Percent change in body weight for (**h**) the H1975 xenograft treatment study and (**i**) the LU1868 PDX treatment study with TEAD inhibitor VT108. (**j-n**) The combination therapies with osimertinib and TEAD inhibitor VT108 are not effective in tumors with complete drug resistance in xenograft models. (**k**) As evaluated by qPCR, YAP-related genes CTGF and CYR61 were decreased in both TEAD inhibitor alone (brown bar) and in combination with osimertinib (red bar) conditions, compared to osimertinib-treated mice (blue bar), verifying YAP/TEAD inhibition. The combination therapy with osimertinib and TEAD inhibitor VT108 did not suppress the relative mRNA expression levels of Bcl-XL, PCNA and KI67 in the resistant-H1975 xenograft treatment study. Compounds were orally administered once daily (PO, QD) throughout the entire study.

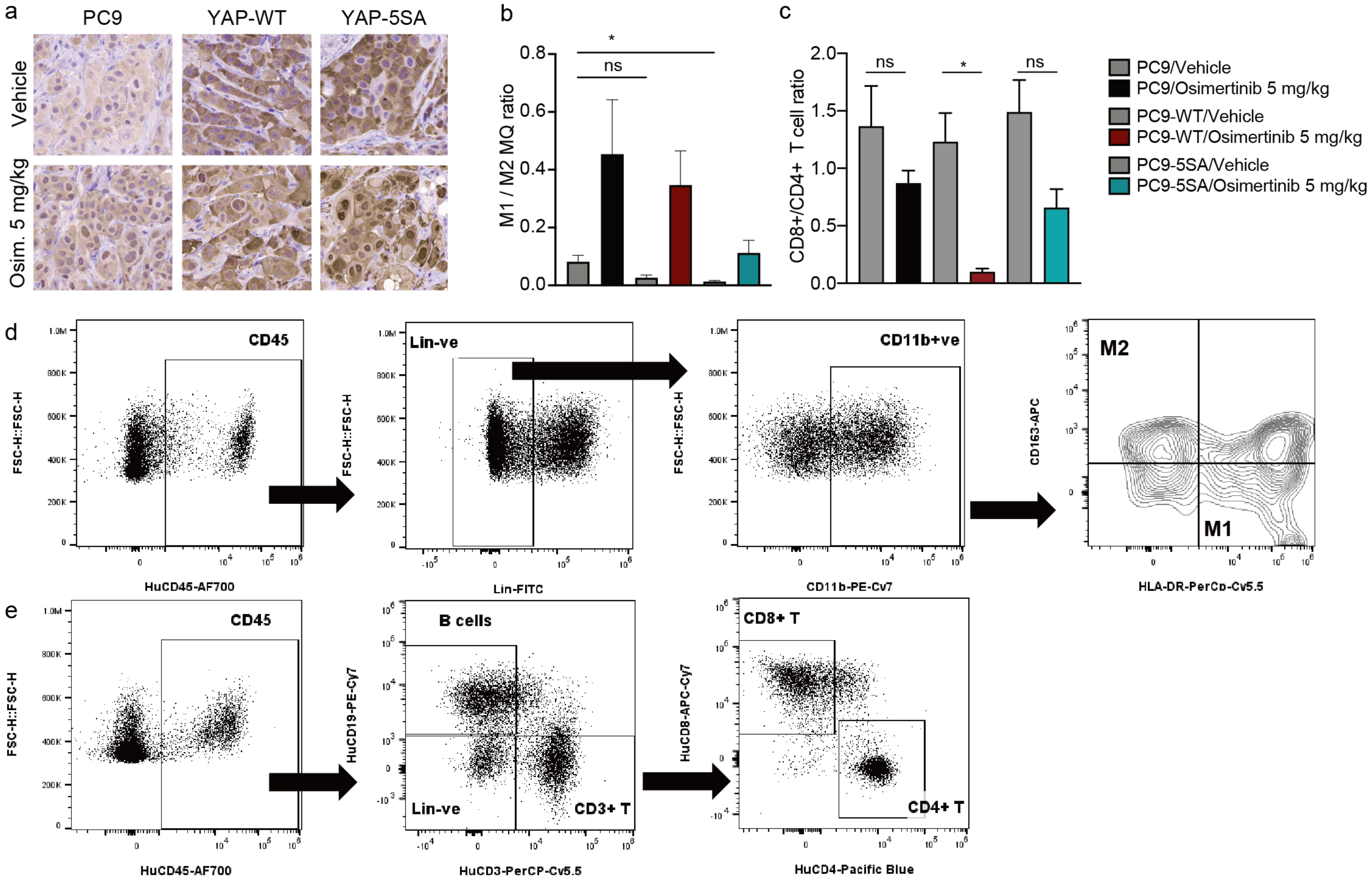

Supplementary Fig. 9. Evaluation of YAP-mediated drug tolerance in immune-competent humanized mice. (**a**) Immunohistochemistry staining for YAP in humanized PC9 mouse model comparing parental cells, cells expressing YAP-WT and cells expressing hyperactive YAP-5SA. (**b**) Ratios of macrophage (M1 / M2) and (**c**) T cell (CD4+ / CD8+) populations at treatment endpoint in 5 mg/kg osimertinib treatment study in humanized PC9 mouse model. Statistical evaluation by unpaired t-test with ns, $p > 0.05$; * $p < 0.05$. (**d-e**) Illustration of the FACS sequential gating strategies for (**d**) M1 and M2 macrophage and (**e**) tumor-infiltrating T cells.

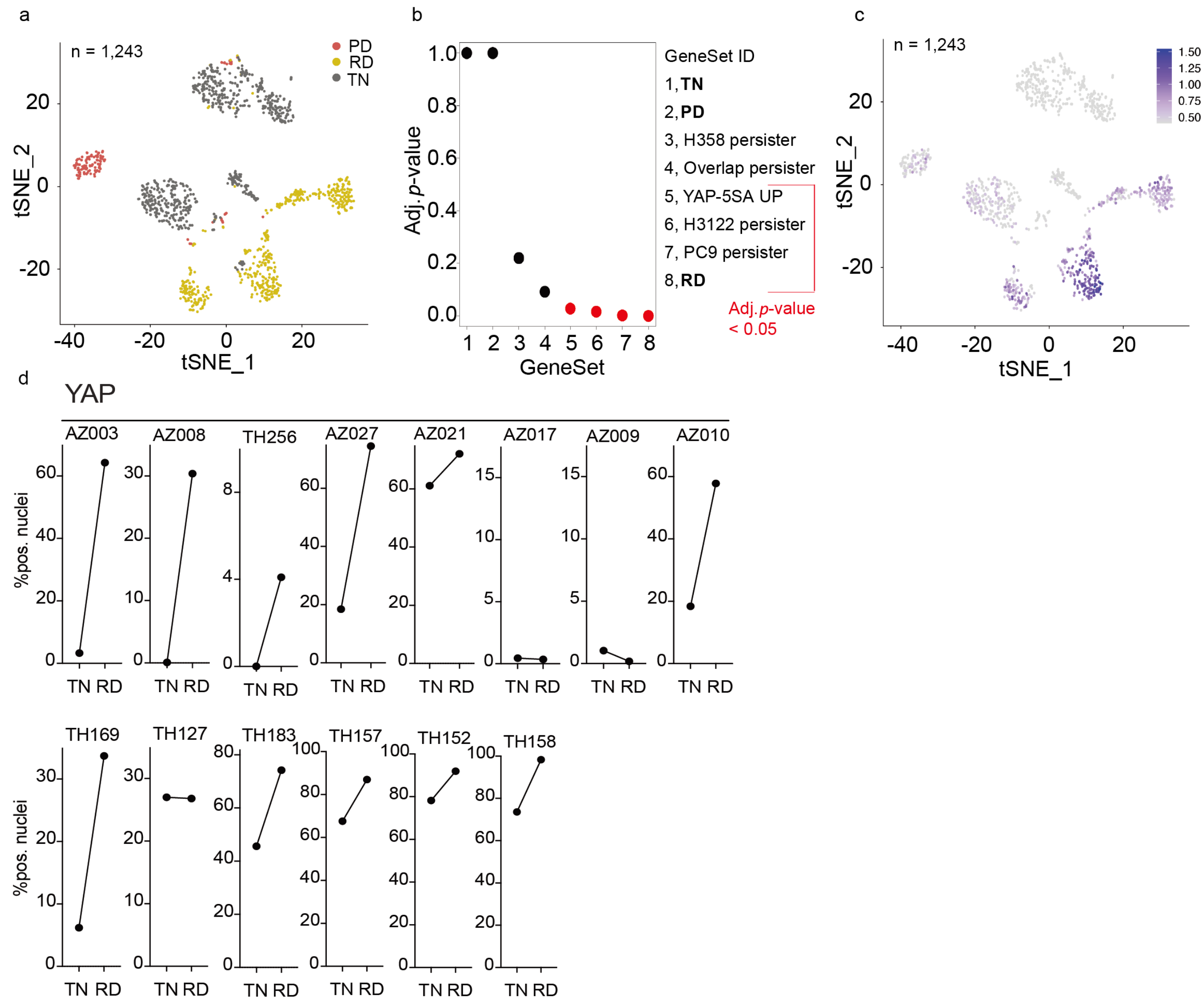

Supplementary Fig. 10. Transcriptional changes and YAP nuclear localization in patient NSCLC specimens.

(**a**) t-stochastic neighbor embedding (t-SNE) plot of all cells derived from primary lung specimens (n = 1,243) and colored by treatment timepoint. (**b**) Permutation analysis for indicated gene sets including bulk RNA sequencing data of persister cell line models as well as transcriptional profiles of scRNAseq data from patient specimens collected across TN, RD and PD. Similarity of expression features was determined in relation to scRNAseq profiled at RD. (**c**) Feature plot highlighting the 50% highest expressors for the YAP gene signature across the t-SNE presentation of all cells derived from primary lung specimens (n = 1,243). The YAP-associated

transcriptional targets that are enriched at RD state (dark purple) according to the state distribution in (a, dark yellow). (**d**) Nuclear levels of YAP in matched patient specimens from TN and RD treatment time points. Increase of nuclear YAP levels in 11/14 (~79 %) matched TN-RD specimens.

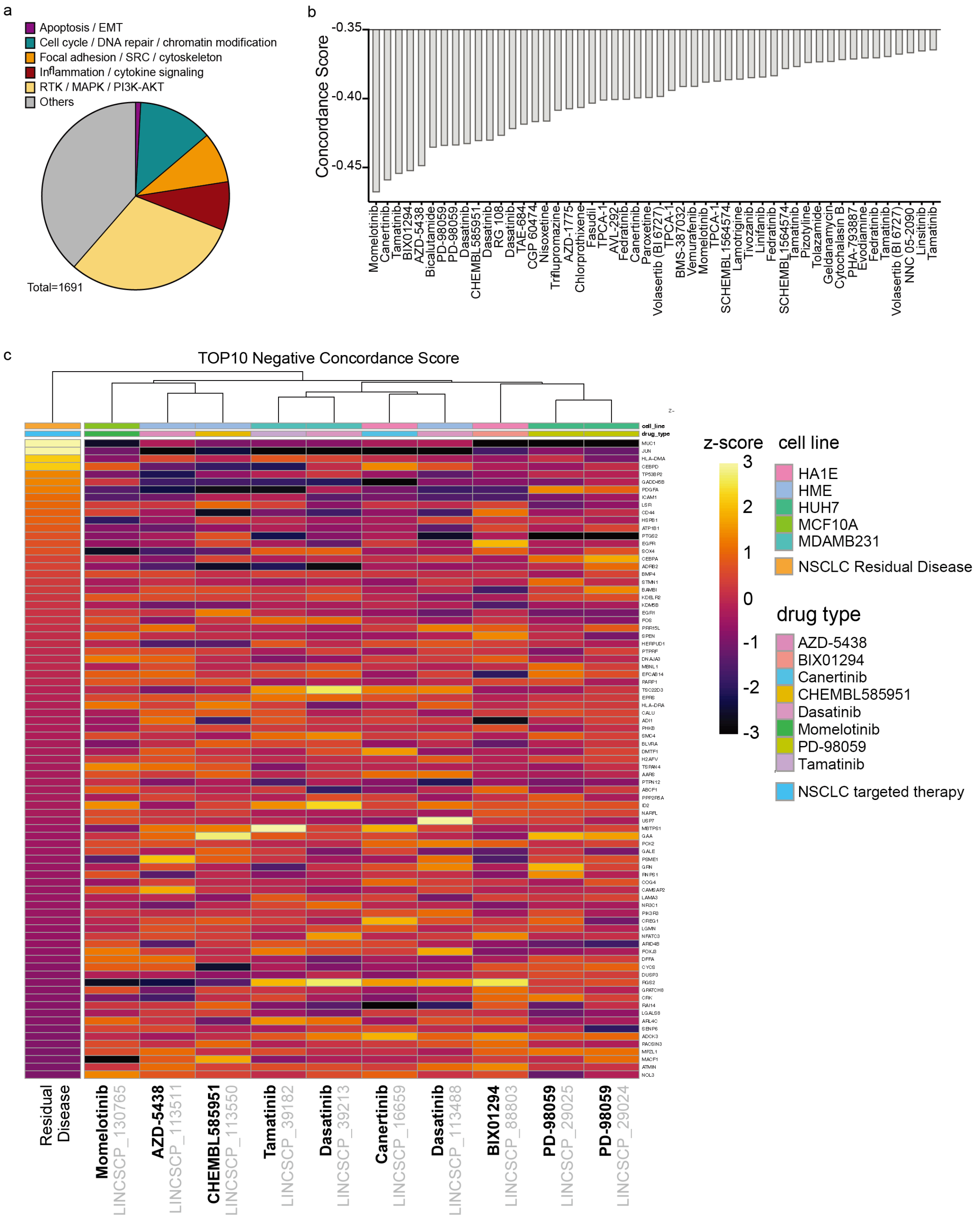
a
Apoptosis / EMT
Cell cycle / DNA repair / chromatin modification
Focal adhesion / SRC / cytoskeleton
Inflammation / cytokine signaling
RTK / MAPK / PI3K-AKT
Others
Total=1691
b
Concordance Score
-0.35
-0.40
-0.45
Momelotinib
Canertinib
Tamatinib
BIX01294
AZD-5438
Bicalutamide
PD-98059
PD-98059
Dasatinib
CHEMBL585951
Dasatinib
RG 108
Dasatinib
TAE-684
CGP 60474
Nisoxetine
Triflupromazine
AZD-1775
Chlorprothixene
Fasudil
TPCA-1
AVL-292
Fedratinib
Canertinib
Paroxetine
Volasertib (BI 6727)
TPCA-1
BMS-387032
Vemurafenib
Momelotinib
TPCA-1
SCHEMBL1564574
Lamotrigine
Tivozanib
Linifanib
Fedratinib
SCHEMBL1564574
Tamatinib
Pizotyline
Tolazamide
Geldanamycin
Cytochalasin B
PHA-793887
Evodiamine
Fedratinib
Tamatinib
Volasertib (BI 6727)
NNC 05-2090
Linsitinib
Tamatinib
c
TOP10 Negative Concordance Score
cell_line
drug_type
z-score
3
2
1
0
-1
-2
-3
cell line
HA1E
HME
HUH7
MCF10A
MDAMB231
NSCLC Residual Disease
drug type
AZD-5438
BIX01294
Canertinib
CHEMBL585951
Dasatinib
Momelotinib
PD-98059
Tamatinib
NSCLC targeted therapy
Residual Disease
Momelotinib LINCSCP_130765
AZD-5438 LINCSCP_113511
CHEMBL585951 LINCSCP_113550
Tamatinib LINCSCP_39182
Dasatinib LINCSCP_39213
Canertinib LINCSCP_16659
Dasatinib LINCSCP_113488
BIX01294 LINCSCP_88803
PD-98059 LINCSCP_29025
PD-98059 LINCSCP_29024

Supplementary Fig. 11. LINCS L1000 concordance analysis across *in vitro* cell lines and clinical NSCLC RD samples.

(**a**) Annotation of LINCS L1000 drug / cell line combinations[44] according to molecular processes. Only LINCS L1000 drug / cell line combinations with significant negative concordance score and target gene information are shown. (**b**) Bar graph of top 50 annotated LINCS L1000 perturbations with negative concordance scores. Drug names annotated by target gene information within the categories "Focal adhesion / SRC / cytoskeleton" and "Inflammation / cytokine signaling" are highlighted in bold. (**c**) Heatmap presentation of drug-induced expression changes by the top 10 annotated LINCS L1000 perturbations (labeled with corresponding unique identifier in gray) across genes differentially regulated in RD scRNAseq specimens.

Supplementary Tables

Supplementary Table 1. Raw counts for counting YAP-knockdown DTP cells (Figure 2k and 2l).

Supplementary Table 2. Raw counts for counting H3122 and H358 DTP cells with VT104 (Supplementary Figure 4b).

Supplementary Table 3. Raw counts for counting PC9 and H358 DTP cells with YAP overexpression (Supplementary Figure 4f and 4g).

Supplementary Table 4. Raw counts for counting EphB1, ACK1, and FAK knockdown in DTP cells (Figure 3d).

Supplementary Table 5. Raw counts for counting FAK- or YAP-knockout cells in PC9 and H358 DTP cells (Figure 3f).

Supplementary Table 6. Raw counts for counting VS-4718 treated H3122, PC9, and H358 DTP cells (Figure 3i).

Supplementary Table 7. Raw counts for counting PC9 and H3122 with Dasatinib and TKI during DTP generation (Supplementary Figure 6g).

Supplementary Table 8. Quantitative PCR raw data and fold change of NCI-H1975 CDX resistant models. (Supplementary Figure 8k).

Supplementary Table 1

knock-down during persister generation

	PC9 cells			H3122 cells		
	siNT	siYAP-1	siYAP-2	siNT	siYAP-1	siYAP-2
**raw counts**	1.10	0.25	0.20	0.32	0.14	0.12
[x10E6 cells]	1.07	0.38	0.23	0.34	0.16	0.13
	1.11	0.38	0.28	0.39	0.16	0.13
**normalized**	1.01	0.23	0.18	0.91	0.40	0.34
	0.98	0.35	0.21	0.97	0.46	0.37
	1.02	0.35	0.26	1.11	0.46	0.37

knock-down at persister stage

	PC9 osimertinib persister			H2228 alectinib persister		
	siNT	siYAP-1	siYAP-2	siNT	siYAP-1	siYAP-2
**raw counts**	1.45	0.87	0.90	0.82	0.34	0.29
[x10E6 cells]	1.31	0.78	1.06	0.85	0.48	0.31
	1.40	0.85	1.01	0.72	0.46	0.24
**normalized**	1.05	0.63	0.65	1.04	0.42	0.36
	0.94	0.57	0.76	1.06	0.61	0.39
	1.01	0.61	0.73	0.90	0.58	0.31

Supplementary Table 2

	H3122 cells			H358 cells		
**VT104**	**0**	**0.1**	**1**	**0**	**0.1**	**1**
**raw counts**	0.024	0.006	0.014	0.31	0.21	0.16
[x10E6 cells]		0.006	0.016	0.37	0.24	0.12
	0.023	0.012	0.010	0.31	0.20	0.16
**normalized**	1.02	0.26	0.60	0.94	0.64	0.49
		0.26	0.68	1.12	0.73	0.36
	0.98	0.51	0.43	0.94	0.61	0.49

Supplementary Table 3

	PC9 cells							
	EV		YAP-WT		YAP-5SA		YAP-S94A	
	DMSO	Osim. 2 µM	DMSO	Osim. 2 µM	DMSO	Osim. 2 µM	DMSO	Osim. 2 µM
**raw counts**	0.82	0.21	0.54	0.20	0.41	0.19	0.55	0.16
[x10E6 cells]	0.72	0.22	0.58	0.20	0.42	0.21	0.59	0.18
	0.80	0.24	0.59	0.21	0.43	0.22	0.55	0.20
**normalized**	1.01	0.46	0.93	0.52	1.10	0.68	1.03	0.34
	0.89	0.36	1.08	0.50	0.93	0.76	0.99	0.44
	1.11	0.34	0.99	0.55	0.97	0.72	0.97	0.48

	H358 cells							
	EV		YAP-WT		YAP-5SA		YAP-S94A	
	DMSO	RMC-4550 1 µM	DMSO	RMC-4550 1 µM	DMSO	RMC-4550 1 µM	DMSO	RMC-4550 1 µM
**raw counts**	0.33	0.17	0.40	0.21	0.18	0.19	0.37	0.18
[x10E6 cells]	0.37	0.15	0.41	0.22	0.20	0.21	0.40	0.18
	0.33	0.16	0.42	0.23	0.22	0.22	0.40	0.17
**normalized**	0.96	0.50	0.98	0.51	0.90	0.95	0.95	0.46
	1.08	0.44	1.00	0.54	1.00	1.05	1.03	0.46
	0.96	0.47	1.02	0.56	1.10	1.10	1.03	0.44

Supplementary Table 4

	PC9 osimertinib persister		H3122 alectinib persister	
	siNT	siEAF	siNT	siEAF
**raw counts**	1.06	0.61	0.28	0.19
[x10E6 cells]	0.96	0.62	0.33	0.18
	1.00	0.62	0.23	0.14
**normalized**	1.05	0.61	1.00	0.67
	0.95	0.62	1.17	0.64
	0.99	0.62	0.83	0.49

Supplementary Table 5

	PC9 cells			H358 cells		
	**CTRL**	**FAK KO**	**YAP KO**	**CTRL**	**FAK KO**	**YAP KO**
**raw counts**	0.85	0.62	0.49	1.32	0.78	0.68
[x10E6 cells]	0.91	0.54	0.51	1.35	0.79	0.72
	0.71	0.47	0.48	1.34	0.75	0.60
**normalized**	1.03	0.75	0.59	0.99	0.58	0.51
	1.10	0.66	0.62	1.01	0.59	0.54
	0.86	0.57	0.58	1.00	0.56	0.45

Supplementary Table 6

	PC9 cells				H3122 cells				H358 cells			
**VS-4718 [µM]**	**0**	**0.5**	**1**	**10**	**0**	**0.5**	**1**	**10**	**0**	**0.5**	**1**	**10**
**raw counts**	1.18	0.80	0.70	0.16	0.39	0.07	0.05	0.03	2.12	0.59	0.49	0.08
[x10E6 cells]	1.14	0.78	0.62	0.15	0.37	0.06	0.06	0.02	2.06	0.59	0.52	0.08
	1.16	0.64	0.59	0.18	0.36	0.07	0.04	0.01	2.18	0.65	0.44	0.07
**normalized**	1.02	0.69	0.60	0.14	1.04	0.19	0.14	0.08	1.00	0.28	0.23	0.04
	0.98	0.67	0.53	0.13	0.99	0.15	0.17	0.06	0.97	0.28	0.25	0.04
	1.00	0.55	0.51	0.16	0.96	0.19	0.12	0.03	1.03	0.31	0.21	0.03

Supplementary Table 7

	PC9 cells		H3122 cells	
**Dasatinib**	**0**	**100**	**0**	**50**
**raw counts**	0.24	0.07	0.29	0.19
[x10E6 cells]	0.25	0.06	0.26	0.19
	0.26	0.07	0.28	0.18
**normalized**	1.03	0.26	0.96	0.69
	1.03	0.24	1.00	0.69
	0.95	0.28	1.04	0.65

Supplementary Table 8

Study ID	Group	Animal No.	Tumor volume at takedown	CTGF Normalized folds (2-ΔΔCt)	CYR61 Normalized folds (2-ΔΔCt)	Bcl-XL Normalized folds (2-ΔΔCt)	PCNA Normalized folds (2-ΔΔCt)	Ki67 Normalized folds (2-ΔΔCt)
VIVACE-20210922 Osi-resistant H1975	Group 1 Vehicle,PO, QD	5315	3534	0.87	0.96	0.96	0.78	0.95
		5362	2775	1.13	1.04	1.04	1.22	1.05
	Group 2 Osimertinib 2.5 mg/kg 10 µL/g PO QD	5303	1951	0.64	1.76	1.55	0.88	0.56
		5322	1607	1.38	2.08	1.27	0.60	0.46
		5323	1425	0.99	1.95	1.62	1.30	0.84
		5340	2083	1.38	1.60	1.20	0.81	0.54
		5344	1524	1.33	2.42	2.14	0.79	0.48
		5352	2651	1.76	3.86	1.73	1.01	0.87
		5382	517	2.05	5.46	1.90	1.56	1.33
		5383	3932	2.13	2.61	2.05	1.47	1.05
	Group 4 VT03989,30 mg/kg, 5 µL/g, PO, QD	5324	2182	0.89	1.07	1.23	1.19	1.07
		5360	3046	0.68	1.06	1.09	1.02	1.03
	Group 6 Osimertinib+VT03989 2.5 mg/kg+30 mg/kg 10 µL/g+5 µL/g PO+PO QD+QD	5325	3058	0.25	1.67	1.78	1.14	0.95
		5330	1782	0.40	2.45	2.14	1.92	1.95
		5336	1154	0.20	0.98	1.35	0.82	0.86
		5348	1476	0.40	1.81	2.50	1.62	1.62
		5351	3124	0.25	0.97	2.22	1.42	1.72
		5391	3567	0.33	1.93	1.79	1.56	1.14
		5395	632	0.40	1.83	2.13	1.39	1.18
