# Supplementary material for: Focal adhesion kinase-YAP signaling axis drives drug-tolerant persister cells and residual disease in lung cancer
Source: Nat Commun. 2024 May 3;15:3741. doi: 10.1038/s41467-024-47423-0 (PMC11068778; doi:10.1038/s41467-024-47423-0)
Supplement: Supplementary file 2 — Reporting Summary [file 41467_2024_47423_MOESM2_ESM.pdf]

nature portfolio

Corresponding author(s): Trever G. Bivona

Last updated by author(s): ~~Nov 6, 2023~~

Reporting Summary

Nature Portfolio wishes to improve the reproducibility of the work that we publish. This form provides structure for consistency and transparency in reporting. For further information on Nature Portfolio policies, see our Editorial Policies and the Editorial Policy Checklist.

Statistics

For all statistical analyses, confirm that the following items are present in the figure legend, table legend, main text, or Methods section.

n/a	Confirmed	
☐	☒	The exact sample size (*n*) for each experimental group/condition, given as a discrete number and unit of measurement
☐	☒	A statement on whether measurements were taken from distinct samples or whether the same sample was measured repeatedly
☐	☒	The statistical test(s) used AND whether they are one- or two-sided<br>*Only common tests should be described solely by name; describe more complex techniques in the Methods section.*
☐	☒	A description of all covariates tested
☐	☒	A description of any assumptions or corrections, such as tests of normality and adjustment for multiple comparisons
☐	☒	A full description of the statistical parameters including central tendency (e.g. means) or other basic estimates (e.g. regression coefficient) AND variation (e.g. standard deviation) or associated estimates of uncertainty (e.g. confidence intervals)
☐	☒	For null hypothesis testing, the test statistic (e.g. *F*, *t*, *r*) with confidence intervals, effect sizes, degrees of freedom and *P* value noted<br>*Give P values as exact values whenever suitable.*
☒	☐	For Bayesian analysis, information on the choice of priors and Markov chain Monte Carlo settings
☒	☐	For hierarchical and complex designs, identification of the appropriate level for tests and full reporting of outcomes
☒	☐	Estimates of effect sizes (e.g. Cohen's *d*, Pearson's *r*), indicating how they were calculated

Our web collection on statistics for biologists contains articles on many of the points above.

Software and code

Policy information about availability of computer code

Data collection

GraphPad Prism 7, QuantStudio 12K Flex Software V1.3, Adobe Illustrator 26.2.1, Fiji ImageJ2, Microsoft Excel, R (version 3.3.1) and deepSNV (v1.18.1), ggplot2 (v.3.3.3), Seurat (v.3.2.2), DESeq, GSEA4.0.1, ANNOVAR, Flow Jo and Kaluza, Aperio Image Scope

Data analysis

RNAseq analysis:
RNA was extracted from snap-frozen tissue or cell pellets. For tissue samples, tissue was minced using a liquid nitrogen-cooled mortar and pestle before RNA extraction. RNA isolation was performed using the RNeasy Mini kit (Qiagen) including an on-column DNase I digestion. RNA quality was assessed by automated electrophoresis using the RNA 6000 Pico Kit and an Agilent 2100 BioAnalyzer (Agilent Technologies, Inc.). RNA was quantified using the Qubit RNA HS Assay Kit and a Qubit 2.0 fluorometer (Thermo Fisher Scientific). Library preparation and paired-end 150bp (PE150, Illumina) RNA sequencing was performed by Novogene (Novogene Corporation, Sacramento, USA). RNA-Seq reads were mapped to the hg19 reference genome using STAR (Spliced Transcripts Align to a Reference, v2.4.2a). The expression level of transcript per million (TPM) reads were quantified using RNA-Seq by Expectation-Maximization algorithm (RSEM v1.2.29). The quantified gene expressions of 26,334 transcripts (including coding genes and non-coding genes) were processed in R studio. Differentially expressed genes between tumor and normal samples were identified using the EdgeR algorithm. Gene set enrichment analysis was done using GSEA 4.0.1 software

scRNA sequencing trajectory:
A BFP-tagged barcode library (Addgene #85968) was delivered via lentiviral infection into isogenic EGFR-mutant PC9-C2 and H1975-B10 cells. Cells were sorted and serially titrated to allow for ~1000 unique barcode groups. After expansion, cells were subjected to 0.1% DMSO or 2 µM osimertinib treatment and frozen down at the indicated timepoints. Cells were thawed, hashed with TotalSeq A anti-human hashtag antibodies (BioLegend), and pooled for single-cell RNA sequencing on the 10X chromium v3 platform (10x Genomics). Cell hash libraries were prepared as specified by BioLegend. Custom barcode amplification was performed by two rounds of PCR. Libraries were sequenced on the NovaSeq Illumina platform (Center for Advanced Technology, UCSF). After NGS sequencing, cells were called with 10X Cell Ranger pipeline

and cell hashes were called using the scEasyMode package in Python. In addition, bulk genomic barcodes were prepared from the same time points used for single-cell RNA sequencing using the Quick Extract gDNA extraction protocol (Lucigen Corporation) and custom barcode amplification primers for NGS library preparation. A custom script for calling genomic barcodes mapping between single-cell genomic barcodes and bulk genomic barcodes collected from the same samples was used to assess population frequency and map onto single-cell transcriptomes. The diversity index was calculated as 1 - Sum_i (pi^2), where pi is the relative abundance of lineage i. The diversity index is at its maximum when all barcode groups are equally abundant and decreases if some barcode groups are enriched and others depleted. The index was scaled by the max possible index given the number of barcode groups which is max (Lineage diversity index) = 1 - n[(1/n)^2] = 1 - 1/n; n: number of barcode groups. Source code for scRNA sequencing and genetic diversity assessment is available here: https://github.com/johnnyUCSF/FH_TB

Whole exome sequencing:
DNA was extracted from snap-frozen cell pellets using the DNeasy Blood & Tissue kit (Qiagen). DNA quality was assessed by automated electrophoresis using the High Sensitivity DNA Kit and an Agilent 2100 BioAnalyzer (Agilent Technologies, Inc.). DNA was quantified using the Qubit dsDNA HS Assay kit and a Qubit 2.0 fluorometer (Thermo Fisher Scientific). Library preparation and paired-end 150bp (PE150, Illumina) DNA sequencing were performed by Novogene (Novogene Corporation, Sacramento, USA). Pair-end fastq files were mapped to the hg19 genome and mutation calling using the SeqMule pipeline64. The VCF files were annotated using ANNOVAR software at a high-performance computing cluster (UCSF Helen Diller Comprehensive Cancer Center). Further analysis of annotated variants was conducted under the RStudio/R environment.

LINCS L1000 Concordance score
The NIH LINCS L1000 database contains gene expression data from cultured human cells treated with small molecule and genetic perturbagens. Level 4 data was sourced from the Gene Expression Omnibus Series GSE70138. Expression data was restricted to small molecule perturbagens and intersected with the residual disease signature (N = 83 genes). Using a previously published computational pipeline69,70, a score for each signature-drug pair was determined using a non-parametric rank-based method that is similar to the Kolmogorov–Smirnov test statistic, where negative scores indicate genes in the ranked drug profile are oppositely regulated in the ranked disease signature. P-values for drug-gene expression profiles were determined by comparing their scores to a distribution of random scores and adjusted with the false discovery rate (FDR; Benjamini-Hochberg, [illegible] = 0.05) method. Metadata and identifiers associated with each perturbagen were sourced from the iLINCS suite. For the upregulated residual disease signature, drug-gene expression profiles were chosen that produce the greatest significant negative score.

For manuscripts utilizing custom algorithms or software that are central to the research but not yet described in published literature, software must be made available to editors and reviewers. We strongly encourage code deposition in a community repository (e.g. GitHub). See the Nature Portfolio guidelines for submitting code & software for further information.

Data

Policy information about availability of data

All manuscripts must include a data availability statement. This statement should provide the following information, where applicable:

- Accession codes, unique identifiers, or web links for publicly available datasets
- A description of any restrictions on data availability
- For clinical datasets or third party data, please ensure that the statement adheres to our policy

This data is available as an NCBI Bioproject under accession number PRJNA766057. For single cell RNA seq analyses of patient specimens, the data is derived from a previously published study and available as an NCBI Bioproject under accession number PRJNA591860. Plasmids, data and codes generated are available by request from the corresponding author.

Research involving human participants, their data, or biological material

Policy information about studies with human participants or human data. See also policy information about sex, gender (identity/presentation), and sexual orientation and race, ethnicity and racism.

Reporting on sex and gender

Participant sex (biological attribute) provided in supplementary tables. No sex- and gender-based analysis was performed, not relevant and outside of scope of current study.

Reporting on race, ethnicity, or other socially relevant groupings

Participant race provided in supplementary tables. No race-based analysis was performed, not relevant and outside of scope of current study.

Population characteristics

Population characteristics relevant in this study:
- Age (adult over 18)
- Lung cancer histology
- Oncogenic driver mutation
- Analysis status
- Treatment history
- Treatment response

Other population characteristics provided but not relevant in this study:
- Smoking history

Recruitment

The patient data is derived from a previously published study and available as an NCBI Bioproject under accession number PRJNA591860.

Ethics oversight

All patients gave informed consent for collection of clinical correlates, tissue collection, research testing under Institutional Review Board (IRB)-approved protocols in a de-identified manner. Patient demographics are listed in Supplementary Table 1. Patient studies were conducted according to the Declaration of Helsinki, the Belmont Report, and the U.S. Common Rule.

Note that full information on the approval of the study protocol must also be provided in the manuscript.

Field-specific reporting

Please select the one below that is the best fit for your research. If you are not sure, read the appropriate sections before making your selection.

☒ Life sciences ☐ Behavioural & social sciences ☐ Ecological, evolutionary & environmental sciences

For a reference copy of the document with all sections, see nature.com/documents/nr-reporting-summary-flat.pdf

Life sciences study design

All studies must disclose on these points even when the disclosure is negative.

Sample size

Sample size of clinical data was determined by access and analysis of all available cases that met criteria. Sample size of mouse experiments were determined based on previous work in the laboratory.

Data exclusions

Details of exclusion criteria based are described in detail in the methods section.

Replication

Studies involving pre-clinical models were performed with two or more biological and/or technical replicates.

Randomization

The mice will be randomly distributed based on tumor size for xenograft studies and grouped into Vehicle and treatment groups. Humanized mice were randomized into treatment and no-treatment groups based on tumor size and donor HLA type.

Blinding

Investigators were blinded during data collection and analysis of mouse tissues.

Reporting for specific materials, systems and methods

We require information from authors about some types of materials, experimental systems and methods used in many studies. Here, indicate whether each material, system or method listed is relevant to your study. If you are not sure if a list item applies to your research, read the appropriate section before selecting a response.

Materials & experimental systems

n/a	Involved in the study	
☐	☒	Antibodies
☐	☒	Eukaryotic cell lines
☒	☐	Palaeontology and archaeology
☐	☒	Animals and other organisms
☐	☒	Clinical data
☒	☐	Dual use research of concern
☒	☐	Plants

Methods

n/a	Involved in the study	
☒	☐	ChIP-seq
☐	☒	Flow cytometry
☒	☐	MRI-based neuroimaging

Antibodies

Antibodies used

For Western blotting, antibodies for phospho-ACK1 (Y284, #3138), Bcl-xL (#2764), phospho-EphB1 (Y324, #3481), EphB1 (#3980), ErbB2 (#4290), ErbB3 (#4754), phospho-FAK (Y397, #8556), FAK (#3285), FGFR1 (#9740), Histone H3 (#9715), Lamin B1 (#12586), phospho-LATS1 (T1069, #8654), LATS1 (#3477), phospho-SRC (Y416, #2101), SRC (#2108), phospho-YAP (S127, #13008), and YAP/TAZ (#8418) were purchased from Cell Signaling Technology. The antibody for phospho-YAP (Y357, #62751) was purchased from Abcam. Antibodies for ACK1 (#sc-28336), FGFR2 (#sc-6930), and GAPDH (#sc-365062) were purchased from Santa Cruz Biotechnology. The antibody for ?-actin (#A2228) was purchased from Sigma-Aldrich. Antibodies were diluted according to the manufacturer's recommendations. For confocal analysis, PLA, and immunoprecipitation, pan-TEAD (#13295) and YAP (#12395) antibodies were purchased from Cell Signaling Technologies and diluted 1:100. For immunohistochemistry, the antibody for YAP (clone H-125, #sc-15407) was purchased from Santa Cruz Biotechnology and diluted 1:150. For immune cell profiling in humanized mice models, fluorochrome-conjugated monoclonal antibodies to the following human antigens were used: CD45-Alexa Fluor 700 (clone 2D1, HI30), CD45-phycoerythrin (PE; clone 2D1, HI30), CD3-PerCp/cy5.5 (clone HIT3a), CD19-PE-cyanine 7 (clone HIB19), CD8-allophycocyanin-cyanine 7 (clone RPA-T8, HIT8a), CD4-Pacific blue (clone OKT4), HLA-DR-PerCp/cy5.5 (clone LN3), CD11b-PE-Cy7 (clone 1CRF-44) (Thermo fisher), CD25-APC (clone CD25-4E3), CD163-APC (clone ebioGH1/61; Thermo fisher). A mouse CD45-FITC (clone 30-F11) antibody was used for gating out murine leukocytes.

Validation

All antibodies were validated with RNAi mediated knockdown approaches or based on the expected changes in those proteins upon treatment with inhibitors. Most of the antibodies used in this study have been extensively used in previously published studies.

Eukaryotic cell lines

Policy information about cell lines and Sex and Gender in Research

Cell line source(s)	Cell lines were purchased from ATCC. Isogenic cell lines are generated based on the parental cell lines from ATCC.
Authentication	Cell lines were previously validated by STR analysis.
Mycoplasma contamination	Cell lines were tested for mycoplasma contamination.
Commonly misidentified lines (See ICLAC register)	N/A

Animals and other research organisms

Policy information about studies involving animals; ARRIVE guidelines recommended for reporting animal research, and Sex and Gender in Research

Laboratory animals	For xenograft studies, 6-8 week old female NOD/SCID mice were used.<br><br>For humanized mouse model, female 3-to-4-week-old NOD. Cg-Prkdcscid Il2rgtm1Wjl/SzJ (NSG) mice, which are suitable for the engraftment of human hematopoietic cells, were housed in microisolator cages under specific pathogen-free conditions in a dedicated humanized mice room in the animal facility. Mice were given autoclaved acidified water and fed a special diet (Uniprim diet).<br><br>An approximately equal number of males and females were used in each arm of the study. Mice were housed with up to 5 mice per cage and separated by sex.<br><br>Mice were examined once daily. At the beginning of each experiment, the animals were weighed, and subsequently, their weight was recorded weekly throughout the duration of the experiment.<br><br>Criteria for Ethical Termination (clinical signs or situations indicating the humane conclusion of the experiment to prevent unnecessary suffering): Mice which showed any one of the signs below were culled by an overdose of anaesthetic and then a terminal bleed (exsanguination) was performed.<br>- If weight loss reaches 10% of their original body weight within 24 hours, or 15% weight loss within 48 hours, or a gradual loss of 15-20% over a longer period.<br>- Persistent hunched posture, piloerection, and labored breathing.<br>- Diarrhea.<br>- Presence of ulcers or signs of infection.<br>- If continuous self-induced trauma is observed.
Wild animals	N/A
Reporting on sex	No sex-based analysis was performed, not relevant and outside of scope of current study.
Field-collected samples	N/A
Ethics oversight	All animal experiments were conducted under IACUC-approved animal protocol no. AN187306-01B or according to the guidelines approved by the IACUC of WuXi AppTec or Crown Bioscience, Inc., following the guidance of the Association for Assessment and Accreditation of Laboratory Animal Care (AAALAC).

Note that full information on the approval of the study protocol must also be provided in the manuscript.

Clinical data

Policy information about clinical studies

All manuscripts should comply with the ICMJE guidelines for publication of clinical research and a completed CONSORT checklist must be included with all submissions.

Clinical trial registration	NCT03433469
Study protocol	The protocol for this study is confidential, but available for review purposes on request.
Data collection	Patients were recruited for this study from July 1 2018 to August 1, 2020. Data were collected for this study at UCSF from July 1, 2018 to December 1, 2020.
Outcomes	The primary outcome of this study is to evaluate the efficacy of osimertinib as neoadjuvant therapy in patients with surgically resectable EGFR-mutant NSCLC. The primary endpoint of the study will be MPR rate defined as ≤10% viable tumor present histologically in the resected tumor specimen. Secondary measures of efficacy are radiographic decrease in maximum tumor diameter, 5-year DFS, 5-year OS, pathological response rate (pCR), and depth of response (DpR). Exploratory endpoints are to evaluate genomic and transcriptional changes on baseline and resected tumor specimens as determined by whole exome sequencing

and RNA sequencing.

Plants

Seed stocks	N/A
Novel plant genotypes	N/A
Authentication	N/A

Flow Cytometry

Plots

Confirm that:

☒ The axis labels state the marker and fluorochrome used (e.g. CD4-FITC).

☒ The axis scales are clearly visible. Include numbers along axes only for bottom left plot of group (a 'group' is an analysis of identical markers).

☒ All plots are contour plots with outliers or pseudocolor plots.

☒ A numerical value for number of cells or percentage (with statistics) is provided.

Methodology

Sample preparation	Apoptotic cell death was detected by flow cytometry using Annexin V and 7-amino-actinomycin (7-AAD) staining.<br><br>For immune analysis in humanized mouse model, single-cell suspensions were prepared. Several 10-color flow cytometry panels were used for immune profiling of both innate and adaptive immune populations in humanized mice and for evaluating immune response after treatment.
Instrument	Apoptotic cells were harvested and resuspended in Annexin V-binding buffer containing 10% Annexin V-FITC and 10% 7-AAD staining solution (Thermo Fisher Scientific).<br><br>For immune cell profiling in humanized mice models, fluorochrome-conjugated monoclonal antibodies to the following human antigens were used: CD45-Alexa Fluor 700 (clone 2D1, HI30), CD45-phycoerythrin (PE; clone 2D1, HI30), CD3-PerCp/cy5.5 (clone HIT3a), CD19-PE-cyanine 7 (clone HIB19), CD8-allophycocyanin-cyanine 7 (clone RPA-T8, HIT8a), CD4-Pacific blue (clone OKT4), HLA-DR-PerCp/cy5.5 (clone LN3), CD11b-PE-Cy7 (clone 1CRF-44) (Thermo fisher), CD25-APC (clone CD25-4E3), CD163-APC (clone ebioGH1/61; Thermo fisher). A mouse CD45-FITC (clone 30-F11) antibody was used for gating out murine leukocytes.
Software	All samples were run on Attune NxT flow cytometer (Thermo fisher), and data were analyzed by Flow Jo and Kaluza software packages.
Cell population abundance	Several 10-color flow cytometry panels were used for immune profiling of both innate and adaptive immune populations in humanized mice and for evaluating immune response after treatment.
Gating strategy	Macrophage populations are defined as CD11b+ cells, with HLA-DR+ for M1 macrophages and CD163+ for M2 macrophages.<br><br>T-cell populations are defined as CD25+/CD3+ cells, with differentiation of CD4+ and cytotoxic CD8+ T-cells.

☒ Tick this box to confirm that a figure exemplifying the gating strategy is provided in the Supplementary Information.
