# Supplementary material for: Focal adhesion kinase-YAP signaling axis drives drug-tolerant persister cells and residual disease in lung cancer
Source: Nat Commun. 2024 May 3;15:3741. doi: 10.1038/s41467-024-47423-0 (PMC11068778; doi:10.1038/s41467-024-47423-0)
Supplement: Supplementary file 3 — Description of Additional Supplementary Files [file 41467_2024_47423_MOESM3_ESM.pdf]

Description of Additional Supplementary Files

Supplementary Data 1

Description: Patient information.

Supplementary Data 2

Description: Gene cluster information for RNA sequencing experiment in EGFR-mutant PC9 cells.

Supplementary Data 3

Description: Differentially expressed genes upon overexpression of hyperactive YAP-5SA.

Supplementary Data 4

Description: YAP-5SA gene set cluster information for scRNAseq trajectory.

Supplementary Data 5

Description: Expression of YAP gene set cluster 3 genes in EGFR-mutant PDO models upon treatment with osimertinib.

Supplementary Data 6

Description: Differentially expressed genes upon 24h combinatorial treatment with 10 μM VT104 or 50 nM dasatinib in DTPs.

Supplementary Data 7

Description: Quantification of YAP nuclear levels in IHC patient specimen.

Supplementary Data 8

Description: LINCS L1000 Concordance score analysis and annotation.
